# The Cucurbitaceae of India: Accepted names, synonyms, geographic distribution, and information on images and DNA sequences

**DOI:** 10.3897/phytokeys.20.3948

**Published:** 2013-03-11

**Authors:** Susanne S. Renner, Arun K. Pandey

**Affiliations:** 1 Systematic Botany and Mycology, University of Munich, Menzingerstr. 67, 80638 Munich, Germany; 2 Department of Botany, University of Delhi, Delhi-110007, India

**Keywords:** Conservation, revised generic boundaries, *Cucumis* wild species, India’s phytogeographic regions, Cucurbitaceae tribal classification, *Trichosanthes*

## Abstract

The most recent critical checklists of the Cucurbitaceae of India are 30 years old. Since then, botanical exploration, online availability of specimen images and taxonomic literature, and molecular-phylogenetic studies have led to modified taxon boundaries and geographic ranges. We present a checklist of the Cucurbitaceae of India that treats 400 relevant names and provides information on the collecting locations and herbaria for all types. We accept 94 species (10 of them endemic) in 31 genera. For accepted species, we provide their geographic distribution inside and outside India, links to online images of herbarium or living specimens, and information on publicly available DNA sequences to highlight gaps in the current understanding of Indian cucurbit diversity. Of the 94 species, 79% have DNA sequences in GenBank, albeit rarely from Indian material. The most species-rich genera are *Trichosanthes* with 22 species, *Cucumis* with 11 (all but two wild), *Momordica* with 8, and *Zehneria* with 5. From an evolutionary point of view, India is of special interest because it harbors a wide range of lineages, many of them relatively old and phylogenetically isolated. Phytogeographically, the north eastern and peninsular regions are richest in species, while the Jammu Kashmir and Himachal regions have few Cucurbitaceae. Our checklist probably underestimates the true diversity of Indian Cucurbitaceae, but should help focus efforts towards the least known species and regions.

## Introduction

Jeffrey’s (1980) and Chakravarty’s (1982) checklists of the Cucurbitaceae of India are now more than three decades old. Over this time, knowledge of the family’s representatives on the Indian continent has grown considerably through botanical exploration, the additions of Naithani (1990), new treatments for Thailand (De Wilde and Duyfjes 2008a) and China (Lu et al. 2011), and revisionary work on genera, such as *Trichosanthes* (De Boer and Thulin 2012) and *Coccinia* (Holstein in press). Added to this, the online availability of taxonomic literature and specimen images, and molecular-phylogenetic studies clarifying natural clade boundaries (e.g., Kocyan et al. 2007; Schaefer et al. 2009; Sebastian et al. 2011; De Boer et al. 2012), have led to many taxonomic and nomenclatural changes. Updating the two checklists of Indian Cucurbitaceae was therefore timely, especially since the Cucurbitaceae include several of the World’s most important vegetables, such as melon (*Cucumis melo*), cucumber (*Cucumis sativus*), watermelon (*Citrullus lanatus*), pumpkin and squash (*Cucurbita* spp.), and bitter gourd (*Momordica charantia*). Having a current list that is linked with molecular data and images may help focus phylogenetic and floristic research on undercollected species, and potentially strengthen conservation efforts.

Here we present a checklist of the Cucurbitaceae of India that treats just over 400 relevant taxon names. For each accepted species, we provide (i) type information including collecting location and herbaria, (ii) synonyms and their types, (iii) information on geographic range inside and outside India, (iv) links to online images of herbarium or living specimens, and (v) brief information on whether or not DNA sequences are available in GenBank at the National Center for Biological Information (http://www.ncbi.nlm.nih.gov ), with citation of relevant studies. DNA sequences today are essential; they help in the quick identification of sterile material via characteristic sequence motifs or “barcoding” (an Asia-focussed example is Li et al. 2011) and are required for evolutionary and biogeographic studies (e.g., Sebastian et al. 2011, De Boer et al. 2012). Even DNA sequences not coming from Indian material can help place the Indian species in context and to recognize if Indian material differs from African or Chinese material going by the same name.

## Materials and methods

Names that have been applied to Indian Cucurbitaceae were taken from Jeffrey (1980, 1981), Chakravarty (1982), and an unpublished compilation provided by Peter Raven (the Missouri Botanical Garden, St. Louis) and Kanchi Gandhi (Harvard University Herbaria, Boston). We also checked floras of neighboring or near-by countries, especially Naithani (1990), the *Flora of China* treatment (Lu et al. 2011), and numerous publications by De Wilde and Duyfjes (cited in our reference list). Information on the types (collector and location) of the 400 names was obtained from protologues, most of them available online. For nomenclatural types from India, we updated the state in which the respective specimen was collected to agree with modern administrative units. Taxonomic or nomenclatural synonyms were obtained by checking relevant post-1980 treatments (cited under the respective genus or species).

Distributions within India (by state) and outside India (by country or continent) were taken mostly from Chakravarty (1946, 1959, 1982), up-dated from floristic treatments, such as Lu et al. (2011) and the work of De Wilde and Duyfjes (e.g., 2004a, b, 2006a, b, c, 2007a, b, 2008a, 2010, and as cited below). The links to images lead to type specimen images from various herbaria or the efloraofindia website (https://sites.google.com/site/efloraofindia/ ). This website has been created for documenting the flora of India and currently has a database of 7500 species and over one million pictures at its e-group links. For each accepted species or relevant synonyms we checked GenBank (http://www.ncbi.nlm.nih.gov ) for sequences and the published studies they are related to.

## Results and discussion

### Comparison with the two 1980s checklists and main causes of name changes

Applying recent taxonomic changes resulted in the acceptance of 94 species. This is almost unchanged from the species number listed in previous checklists (Jeffrey 1980: 90 species; Chakravarty 1982: 100 species). A species no longer included is *Zehneria wallichii* from central Myanmar. Newly added species include *Trichosanthes khasiana* and *Trichosanthes quinquangulata*. Compared to 1980, generic concepts have changed considerably, with many species names having been moved, especially in the genera *Cucumis* and *Zehneria*, and formerly monotypic genera having been merged (Schaefer and Renner 2011b). Genera no longer accepted are *Biswarea* (=*Herpetospermum*), *Cucumella* (=*Cucumis*), *Dicoelospermum* (=*Cucumis*), *Edgaria* (=*Herpetospermum*), *Gymnopetalum* (=*Trichosanthes*), *Mukia* (=*Cucumis*), *Neoluffa* (= *Siraitia*), *Praecitrullus* (= *Benincasa*), and *Sechium* (=*Sicyos*). All these changes are based on molecular-phylogenetic results, cited under the respective species. *Melothria* in its modern circumscription is confined to the New World and does not occur in India. Its two Indian species have been moved to *Cucumis* and *Solena*.

Compared to other tropical regions of the size of India, for example, Brazil, the addition of new species records over the past 30 years has lagged behind. We suspect that many species new for India are awaiting discovery in the field and in yet unidentified herbarium material. Since Indian herbaria are reluctant to send out loans, their material probably is understudied.

### Natives, endemics, cultivated species, and status of DNA sequencing

Of the species of Cucurbitaceae in India, at least nine are introduced cultivated vegetables from Central and South America or Africa (*Citrullus lanatus*, *Cyclanthera pedata***,** *Kedrostis foetidissima***,** *Sicyos edulis*, and five species of *Cucurbita*). Of the native species, ten are endemic: *Cucumis indicus* (Kerala, Maharashtra), *Cucumis ritchiei* (Karnataka, Kerala, Maharashtra, Punjab, Tamil Nadu), *Cucumis setosus* (Gujarat, Karnataka, Madhya Pradesh, Maharashtra, Rajasthan), *Cucumis silentvalleyi* (Kerala), *Momordica sahyadrica* (Kerala), *Solena amplexicaulis* (Tamil Nadu, Karnataka, Kerala), *Trichosanthes anaimalaiensis* (Andaman and Nicobar Islands, Andhra Pradesh, Arunachal Pradesh, Karnataka, Kerala, Maharashtra, Tamil Nadu, Tripura), *Trichosanthes khasiana*, *Zehneria hookeriana* (Tamil Nadu), and *Zehneria maysorensis* (Kerala). Clearly, Kerala is the state with the highest number of endemics, followed by Tamil Nadu. The most species-rich Cucurbitaceae genera in India are *Trichosanthes* with 22 species, *Cucumis* with 11 (all but two wild), *Momordica* 8, and *Zehneria* with 5.

**Figure 1.**
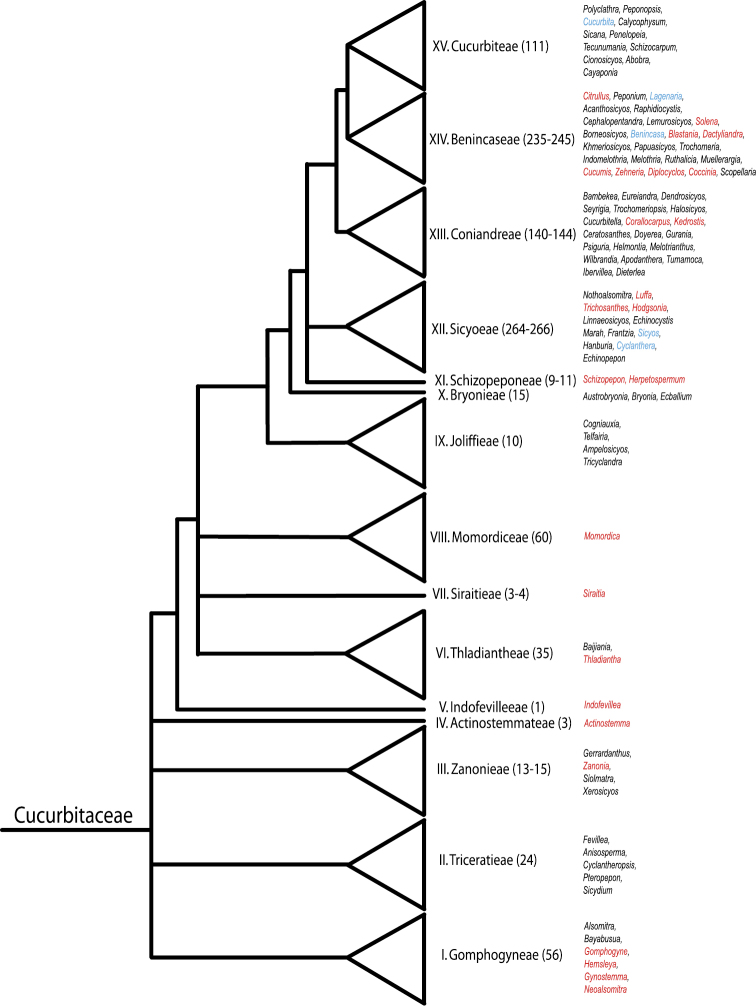
Tribal classification of the Cucurbitaceae with native Indian genera highlighted in red, cultivated ones in blue. Modified from Schaefer and Renner (2011a, b).

While 86 native species, including just ten endemics, may not be large numbers, India harbors an exceptional range of tribes as seen in Fig. 1, which shows the placement of the native Indian genera on a Cucurbitaceae family tree with the family’s current tribal classification (Schaefer and Renner 2011b). Many of the Indian species, such as *Actinostemma*, *Gynostemma*, *Hemsleya*, *Indofevillea*, *Momordica* and *Siraitia* belong to old and phylogenetically isolated lineages. This is known because 79% of the Cucurbitaceae species occurring in India have been sequenced for one or more genetic markers. Cucumber and melon, which originate in India, both have had their genomes completely sequenced (Huang et al. 2009; García-Mas et al. 2012), and many have been included in family-wide phylogenetic analyses (Kocyan et al. 2007; Schaefer et al. 2009; Schaefer and Renner 2011b). The currently 20 species without any DNA sequences in GenBank may be found by searching our checklist for “no published sequences available.”

### Floristic distribution within India and disjunctions between Africa and India

The highest number of species is known from the northeast and peninsular India (Kerala, Karnataka, Tamil Nadu, Andhra Pradesh), the lowest from the Jammu Kashmir and Himachal regions of Western Himalaya. Especially interesting from a phytogeographic standpoint are species ranging from Africa to India, such as *Coccinia grandis*, *Blastania cerasiformis*, *Corallocarpus conocarpus*, *Corallocarpus epigaeus*, *Corallocarpus schimperi*, *Cucumis prophetarum*, *Dactyliandra welwitschii*, *Luffa echinata***,** *Momordica cymbalaria*, and *Zehneria thwaitesii*. The genera *Diplocyclos* and *Kedrostis* also both have species in East Africa and India, but apparently not individual species spanning both continents. These disjunctions would be interesting to study with molecular methods, which might allow inferring arrival times in India.

**Table 1.** Genera and species of Cucurbitaceae in India (94 total).

**Genera**	**Number of species**
*Actinostemma*	1
*Benincasa*	2
*Blastania*	2
*Bryonia*	2
*Citrullus*	2
*Coccinia*	1
*Corallocarpus*	3
*Cucumis*	11
*Cucurbita*	5 (all cultivated)
*Cyclanthera*	1
*Dactyliandra*	1
*Diplocyclos*	1
*Gomphogyne*	1
*Gynostemma*	1
*Hemsleya*	1
*Herpetospermum*	3
*Hodgsonia*	1
*Indofevillea*	1
*Kedrostis*	2
*Lagenaria*	1
*Luffa*	4
*Momordica*	8
*Neoalsomitra*	1
*Schizopepon*	3
*Sicyos*	1
*Siraitia*	1
*Solena*	3
*Thladiantha*	2
*Trichosanthes*	22
*Zanonia*	1
*Zehneria*	5

## Conclusion

One of the great technical advances of recent years that are positively affecting taxonomy is the easy exchange of photos. Even simple snap shots of living plants (and certainly type images) greatly facilitate deciding the identity of a particular plant, and we hope that our links to the efloraofindia (https://sites.google.com/site/efloraofindia/ ) will proof useful. The greatest caveat concerning our checklist is that the geographic range information inside India is not directly based on specimens, but is more or less copied from Chakravarty (1982) and thus surely incomplete. It is to be hoped that the digization of Indian material in the future will help achieve a deeper study of the Cucurbitaceae of India.

## Checklist

**1. *Actinostemma tenerum*** Griff., J. Asiat. Soc. Bengal 23(7): 643–644. 1854.

Syntypes: India, Meghalaya, Khasia Hills, *Griffith 2523* (K, W); India, Sadiya, upper Assam, also on Khasia Hills, *T.E. Cantor*s.n. (K).

Distribution in India: Arunachal Pradesh, Assam, Bihar, Meghalaya, Mizoram, Uttar Pradesh, West Bengal.

Distribution outside India: Bangladesh, Vietnam, Laos, Cambodia, Russia, China, Taiwan, Korea, and Japan (Schaefer and Renner 2011a).

Images: *Griffith* syntype: http://herbarium.univie.ac.at/database/detail.php?ID=63181

http://apps.kew.org/herbcat/getImage.do?imageBarcode=K000742924

GenBank: Sequences from Kocyan et al. (2007), e.g., DQ491007, DQ469135.

Comments: *Actinostemma* comprises two other species, both in China. Based on genetic data, this is an isolated ancient lineage (Schaefer and Renner 2011a, b; see also our Fig. 1). Ali Khan (2002) discusses the species’ occurrence in Uttar Pradesh.

**2. *Benincasa fistulosa*** (Stocks) H.Schaef. & S.S.Renner, Taxon 60: 133. 2011.

*Citrullus fistulosus* Stocks, Hooker’s J. Bot. Kew Gard. Misc. 3: 74, t. 3. 1851. *Citrullus vulgaris* Schrad. ex Eckl. & Zeyh. var. *fistulosus* (Stocks) J.L.Stewart, Punjab Pl. 96. 1869.

*Praecitrullus fistulosus* (Stocks) Pangalo, Bot. Zhurn. S.S.S.R. 29: 203. 1944.

*Colocynthis citrullus* (L.) Kuntze var. *fistulosus* (Stocks) Chakrav., Rec. Bot. Surv. India 17(1): 116. 1959.

*Citrullus lanatus* (Thunb.) Matsum. & Nakai var. *fistulosus* (J.L.Steward) Babu, Herb. Fl. Dehra Dun 194. 1977, invalid name because Babu erred in the basionym he cited.

*Citrullus lanatus* (Thunb.) Matsum. & Nakai var. *fistulosus* (Stocks) Chakrav., Fasc. Fl. India 11: 23. 1982, nom. illeg. isonym.

Type: Pakistan [India], Kurrachee, Sinde, 1 Sep. 1850, *Stocks* s.n. (K).

Distribution in India: Punjab, Rajasthan, Uttar Pradesh.

Distribution outside India: Introduced (?) in tropical Africa.

GenBank: Sequences from Dane and Lang (2004) and Kocyan et al. (2007), e.g., DQ536719, DQ648185, AY522525.

Comments: Cultivated in India and Pakistan as a vegetable. The origin of *Benincasa fistulosa* is unclear, and the species is currently only known in cultivation.

**3. *Benincasa hispida*** (Thunb.) Cogn. in A. & C. DC., Monog. Phan. 3: 513. 1881.

*Cucurbita hispida* Thunb., Nov. Acta Regiae Soc. Sci. Upsal. 4: 38. 1783.

*Benincasa pruriens* (Parkinson) W.J.de Wilde & Duyfjes forma *hispida* (Thunb.) W.J.de Wilde & Duyfjes, Sandakania 17: 47. 2008.

Type: Japan, *Thunberg 22775* (UPS, IDC microfiche). *Benincasa cerifera* Savi, Bibliot. Ital. (Milan) 9: 158-165, f. a-g. 1818.

Type: China, cult. in the Pisa botanical garden (herbarium?).

Distribution in India: Cultivated in tropical and subtropical regions of India.

Distribution outside India: Pakistan. India, Pakistan, Malayasia, Eastern Australia, Polynesia, China & Japan. Wild origin unclear.

Images: http://plants.usda.gov/java/profile?symbol=BEHI3

Efloraofindia at https://sites.google.com/site/efloraofindia/species/a---l/cl/cucurbitaceae/benincasa/benincasa-hispida

GenBank: Sequences from Kocyan et al. (2007), e.g., DQ282075, DQ282074.

Comments: *Benincasa* comprises only the two species that occur in India (Schaefer and Renner 2011a; see our Fig. 1 for the phylogenetic position of the tribe Benincaseae). Nicolson and Fosberg (2003) have argued that the name *Benincasa hispida* (Thunb.) Cogn. does not need to be replaced by *Cucurbita pruriens* Parkinson (J. Voy. South Seas 44 (1773), while De Wilde and Duyfjes (2008b) maintain that the oldest available name for this species is *Cucurbita pruriens*, hence *Benincasa pruriens* (Parkinson) W.J. de Wilde & Duyfjes.

**4.** ***Blastania cerasiformis*** (Stocks) A.Meeuse, Bothalia 8: 12. 1962.

*Ctenolepis cerasiformis* (Stocks)Hook.f., Fl. Trop. Afr. 2: 558. 1871.

*Bryonia fimbristipula* Fenzl ex Stocks, Hooker’s J. Bot. Kew Gard. Misc. 4: 149. 1852, nom. inval. pro syn. of *Zehneria cerasiformis* Stocks

*Blastania fimbristipula* Kotschy & Peyr., Pl. Tinn. 15. t. 7. 1867.

*Melothria fimbristipula* (Kotschy & Peyr.) G. Roberty, Bull. I.F.A.N., Ser. 16:795. 1954.

*Zehneria cerasiformis* Stocks, Hooker’s J. Bot. Kew Gard. Misc. 4: 149. 1852.

Syntype: Africa, Sudan, Blue Nile Province, Jebal Arashkol *Kotschy* 205 (CAL 2 sheets, photos available from SSR, K); Pakistan, *Stocks* 29 (K).

Distribution in India: Wild on wastelands in Gujarat.

Distribution outside India: Old World tropics from Mauritania & Senegal east to Pakistan and in E. Africa south to Transvaal.

Image: http://www.zimbabweflora.co.zw/speciesdata/species.php?species_id=157060

GenBank: Sequences from Kocyan et al. (2007), e.g., DQ535797, DQ536803.

Comment: The genus name *Blastania* Kotschy et Peyritsch was published in July 1867 (the full publication is online at the Biodiversity Heritage Library) and has priority over *Ctenolepis* J. D. Hooker in Bentham et J. D. Hooker, Gen. 1: 832. Sep 1867. Jeffrey (1980) and Chakravarty (1982) both list *Blastania cerasiformis* (under *Ctenolepis*) in their checklists, but we have not seen Indian specimens.

**5. *Blastania garcinii*** (Burm.f.) Cogn. in A. & C. DC., Monogr. Phan. 3: 629. 1881.

*Ctenolepis garcinii* (Burm.f.) Benth. & Hook.f., Gen. Pl. 1(3): 832. 1867.

*Bryonia garcinii* (Burm.f.) Willd., Sp. Pl. 4(1): 623. 1805 (as *garcini*).

*Sicyos garcinii* Burm. f., Fl. Ind. 211 (err. typ. 311). 1768.

Type: India, Tamil Nadu, Chennai (formerly Madras), Tuticorin, *Garcin* s.n. (G) fide Jeffrey, 1980.

Distribution in India: Andhra Pradesh, Delhi, Gujarat, Haryana, Karnataka, Kerala, Madhya Pradesh, Maharashtra, Odisha, Punjab, Rajasthan, Tamil Nadu, Uttar Pradesh.

Distribution outside India: Sri Lanka.

Image: Nothing reliable found.

GenBank: No published sequences available.

Comments: *Blastania* includes *Blastania cerasiformis* from India and west to tropical Africa, *Blastania garcinii* from India and Sri Lanka, and a third species in Madagascar.

**6.** ***Bryonia aspera*** Steven ex Ledeb., Fl. Ross. 2:140. 1843.

Lectotype: Northern Caucasus, Narzan, *Bieberstein* (LE), designated by Jeffrey (1969).

Distribution in India: NW India: Jammu (Upper Chenab Valley), Himachal Pradesh (Chamba, Lahul-spiti).

Distribution outside India: Turkey, Iran, Georgia, Armenia, Azerbaijan, Turkmenistan, Norther Afghanistan, Pakistan.

Image: Nothing reliable found.

GenBank: Sequences from Volz and Renner (2009), e.g., EU683747, EU683740.

Comment: This was treated as *Bryonia dioica* Jacq. by Chakravarty (1982), but that species does not occur as far east as India, ranging instead from Spain south to Algeria and Morocco, Sardinia, Corsica, and the Greek Peninsula and east to mid-Poland; a distribution map with all species of *Bryonia* is provided by Volz and Renner (2009).

**7. *Bryonia monoica*** Aitch. & Hemsl., Trans. Linn. Soc. London, Bot. 3(1): 65. 1888.

Type: Afghanistan, Badghis, *Aitchison 339* (CAL photo available from SSR, K).

Distribution in India: Probably near the Pakistani border.

Distribution outside India: Kazakhstan, Uzbekistan, Kirgizstan, Turkmenistan, Afghanistan, Iran, Pakistan.

Image: Nothing reliable found.

GenBank: Sequences from Volz and Renner (2009), e.g., EU096421, EU096419.

Comment: Chakravarty (1982) treated this under the name *Bryonia multiflora* Boiss. & Heldr., but that species occurs instead in Turkey, Iran, Iraq and Syria (Jeffrey 1969; Volz and Renner 2009).

**8. *Citrullus colocynthis*** (L.) Schrad., Linnaea 12: 414. 1838. *Cucumis colocynthis* L., Sp. Pl. 2: 1011. 1753.

*Colocynthis vulgaris* Schrad., Ind. Sem. 1:fig. 99. 1950.

Type: Not designated.

Distribution in India: Andhra Pradesh, Assam, Bihar, Jahrkhand, Delhi, Goa, Gujarat, Karnataka, Kerala, Maharashtra, Odisha, Punjab, Rajasthan, Tamil Nadu, Uttar Pradesh.

Distribution outside India: Afghanistan, Myanmar, Pakistan, Sri Lanka, west to the Sahara (Lybia) and Sahel region.

Images: See efloraof India at https://sites.google.com/site/efloraofindia/species/a---l/cl/cucurbitaceae/citrullus/citrullus-colocynthis

GenBank: Sequences from Kocyan et al. (2007), e.g., DQ536649, DQ535791.

Comments: *Citrullus colocynthis*, or colocynth, is a perennial growing wild on sandy soils in deserts areas in Western and Central India. Many authors have treated Herb. Linn. No. 1152.1 (LINN) as the type. However, this collection lacks the relevant *Species Plantarum* number and was a post-1753 addition to the herbarium; it is not original material for the name (Jarvis, 2007).

**9. *Citrullus lanatus*** (Thunb.) Matsum. & Nakai, Cat. Sem. & Spor. Hort. Bot. Univ. Imp. Tokyo 1916: 30. 1920 (“1916”).

*Momordica lanata* Thunb., Prodr. Pl. Cap. 13. 1794.

Type: South Africa, Cape Province, *Thunberg* s.n. (UPS).

*Cucurbita citrullus* L., Sp. Pl. 2: 1010. 1753.

Type: “Habitat in Apulia, Calabria, Sicilia”; lectotype not designated.

*Citrullus vulgaris* Schrad. ex Eckl. & Zeyh., Enum. Pl. Afric. Austral. 2: 279. 1836.

Type: Not known fide De Wilde and Duyfjes (2010).

Distribution in India: Andaman & Nicobar Islands, Assam, Bihar, Jahrkhand, Delhi, Gujarat, Karnataka, Madhya Pradesh, Maharashtra, Punjab, Rajasthan, Tamil Nadu, Tripura, Uttar Pradesh, Uttarakhand, West Bengal.

Distribution outside India: Nepal, Pakistan; native to tropical Africa.

Images: The Thunberg holotype can be seen here: http://130.238.83.220/botanik/browserecord.php?-action=browse&-recid=371376

http://www.flowersofindia.net/catalog/slides/Watermelon.html

See also efloraofindia at https://sites.google.com/site/efloraofindia/species/a---l/cl/cucurbitaceae/citrullus/citrullus-lanatus

GenBank: Several hundred sequences.

Comments: The watermelon was probably domesticated in northern Africa (Wasylikowa and van der Veen 2004). The extent of its native range is unclear.

**10. *Coccinia grandis*** (L.) Voigt, Hort. Suburb. Calcutt. 59. 1845.

*Bryonia grandis* L., Mant. Pl. 126. 1767.

Type: India, without location, Herb. Linn. No. 1153.2 (LINN).

*Bryonia alceifolia* [sphalm. *alceaefolia*] Willd. in Rottler, Neue Schriften d. Ges. Naturf. Freunde Berlin 4: 223. 1803.

Type: India, Tamil Nadu, Tiruchinapally [Tiruchirappalli], Nov. 1793, *Rottler* s.n. (K).

*Coccinia indica* Wight & Arn., Prodr. Fl. Ind. Orient. 1: 347. 1834, nom. superfl. & illeg. for *Bryonia grandis* L.

*Coccinia wightiana* M.Roem., Syn. Pepon.: 93. 1846.

Syntypes: India, Chennai, *Wallich Cat. 6711a* [D.Klein, B.Heyne or J.P.Rottler] in Herb. Madras s.n. (Paralectotype: E00174668); Nepalry, *Wallich Cat. 6711b* and 6711*e, R.Wight 1124* (Paralectotype: E00174667); Negapatam, *R.Wight 1124* (Lectotype, designated by Holstein, 2012: E00174666); *R.Wight 1124* (Paralectotype: NY, digital image).

*Coccinia cordifolia* (L.) Cogn. var. *wightiana* (M.Roem.) Cogn. in A. & C. DC., Monogr. Phan. 3: 531. 1881.

*Coccinia grandis* (L.) Voigt var. *wightiana* (M.Roem.) Greb. in R. Mansfeld & J. Schultze-Motel, Verz. Landwirtsch. u. Gaertn. Kulturpfl. 2: 929. 1986.

*Cephalandra indica* Naudin var. *palmata* C.B. Clarke, Fl. Brit. India 2: 621. 1879, nom. & stat. nov.

Distribution in India: Distributed in plains of India, ascending c. 300 m in Peninsular India; Andaman & Nicobar Islands, Andhra Pradesh, Assam, Bihar, Jharkhand, Goa, Gujarat, Himachal Pradesh, Karnataka, Kerala, Lakshadweep, Madhya Pradesh, Chhattisgarh, Maharashtra, Manipur, Odisha, Rajasthan, Tamil Nadu, Tripura, Uttar Pradesh, Uttarakhand, West Bengal.

Distribution outside India: Africa, China, Japan, Malesia, Myanmar, Pakistan, Sri Lanka.

Images: http://www.flowersofindia.net/catalog/slides/Ivy%20Gourd.html

http://apps.kew.org/herbcat/getImage.do?imageBarcode=K000742794

Efloraofindia at https://sites.google.com/site/efloraofindia/species/a---l/cl/cucurbitaceae/coccinia/coccinia-grandis

GenBank: Sequences from Holstein and Renner (2011), e.g., HQ608245, HQ608458.

Comments: The genus *Coccinia* has 35 species, all but *Coccinia grandis* in Africa south of the Sahara (Holstein, in press). In India, *Coccinia grandis* has been used in traditional medicine for hundreds of years (Nadkarni and Nadkarni 1976; Ramachandran and Subramaniam 1983).

**11. *Corallocarpus conocarpus*** (Dalzell & A.Gibson) Hook.f. ex C.B. Clarke, Fl. Brit. India 2: 628. 1879 (as *conocarpa*).

*Aechmandra conocarpa* Dalzell & A.Gibson, Bombay Fl. 100. 1861.

Type: India, Maharashtra, Bombay, Gujrat near Malpor and Gundar, *Dalzell 39* (K).

Distribution in India: Gujarat, Karnataka, Maharashtra, Rajasthan, Tamil Nadu.

Distribution outside India: Pakistan (fide the *Flora of Pakistan*, http://www.tropicos.org/Name/50326465?projectid=32 , the species occurs also in Central Africa)

Image: http://apps.kew.org/herbcat/detailsQuery.do?imageId=375483&pageCode=3&presentPage=3&queryId=4&sessionId=CE49DA6B1178914C12C060C6D319E224&barcode=K000592620

GenBank: No published sequences available.

Comments: *Corallocarpus* has two species in Madagascar, eight in Africa (Schaefer and Renner 2011a), and three that supposedly range from India to tropical East Africa. Chakravarty (1982) accepted four species for India, *Corallocarpus conocarpus*, *Corallocarpus epigaeus*, *Corallocarpus gracilipes*, and *Corallocarpus palmatus*, while Jeffrey (1980) considered the latter two names synonyms of *Corallocarpus epigaeus* as do we, but also accepted *Corallocarpus schimperi* for India.

**12. *Corallocarpus epigaeus*** (Rottler) Benth. & Hook.f. ex C.B. Clarke, Fl. Brit. India 2: 628. 1879 (as *epigaea)*.

*Bryonia epigaea* Rottler, Neue Schriften d. Ges. Naturf. Freunde Berlin 4: 212. 1803.

*Aechmandra epigaea* (Rottler) Arn., J. Bot. 3: 274. 1841.

*Rhynchocarpa epigaea* (Rottler) Naudin, Ann. Sci. Nat., Bot. sér. 4, 16: 178. 1862. Syntypes: Peninsular India, *Klein 395 & 771* (B-W), *Rottler 3531* (HBG), *Rottler* (K).

*Rhynchocarpa epigaea* var. *gracilipes* Naudin, Ann. Sci. Nat., Bot. sér. 4, 16: 179. 1862. *Corallocarpus gracilipes* (Naudin) Cogn. in A. & C. DC., Monogr. Phan. 3: 656. 1881.

Type: India, *J. Lepine* (P).

*Corallocarpus palmatus* Cogn. in A. & C. DC., Monogr. Phan. 3: 648. 1881.

Type: India, Gujarat [Gujerat] near Malpor and Gundar, *Dalzell* s.n. (K).

Further synonyms are listed in Jeffrey (1967).

Distribution in India: Andhra Pradesh, Assam, Bihar, Gujarat, Karnataka, Kerala, Madhya Pradesh, Maharashtra, Punjab, Rajasthan, Tamil Nadu, Uttar Pradesh, West Bengal.

Distribution outside India: Baluchistan, Pakistan, Sri Lanka; tropical East Africa, Sudan.

Image: http://www.arkive.org/corallocarpus/corallocarpus-epigaeus/image-G117835.html

Efloraofindia at https://sites.google.com/site/efloraofindia/species/a---l/cl/cucurbitaceae/corallocarpus/corallocarpus-epigaeus

GenBank: AM981182 from an unpublished paper.

Comments: The species is used as an anthelmintic (Chopra et al. 1956).

**13. *Corallocarpus schimperi*** (Naudin) Hook.f., Fl. Trop. Afr. 2: 567. 1871.

*Rhynchocarpa schimperi* Naudin, Ann. Sc. Nat., sér. 4, 16: 180. 1862.

Type: Ethiopia, Sera-Walqua, *Schimper 413* (P).

*Corallocarpus velutinus* (Dalzell & A.Gibson) Hook.f. ex C.B. Clarke, Fl. Brit. India 2(6): 628. 1879.

*Aechmandra velutina* Dalzell & A.Gibson, Bombay Fl. 200. 1861.

Type: W. Pakistan, *Dalzell 41* (K).

*Corallocarpus courbonii* (Naudin) Cogn. A. & C. in DC. Monogr. Phan. 3: 655. 1881.

Type: A plant cultivated in Paris from seeds sent from Ethiopia, *A. Courbon 334* (P P00346198, http://plants.jstor.org/search?plantName=Rhynchocarpa%20courbonii ).

Distribution in India: Unclear.

Distribution outside India: Pakistan and tropical East Africa and Arabia

Image: See *Flora of Pakistan*: http://www.tropicos.org/Name/9201617?projectid=32

GenBank: No published sequences available.

Comments: The supposed three species of *Corallocarpus* in India are in urgent need of taxonomic study.

**14.** ***Cucumis hystrix*** Chakrav., J. Bombay Nat. Hist. Soc. 50(4): 896. pl. 6. 1952.

Type: India, Meghalaya [earlier in Assam], Garo Hills, Tura Mountain, alt. 3000 ft; November 1929; *N.E. Parry 859* (K).

*Cucumis muriculatus* Chakrav., J. Bombay Nat. Hist. Soc. 50(4): 896. 1952.

Type: Myanmar, Ruby Mines District, Oct. 1912, *J. H. Lace 6325* (E), here synonymized by Kirkbride (1993).

Distribution in India: Arunachal Pradesh, Assam, Meghalaya, Mizoram.

Distribution outside India: Myanmar, N and W Thailand, SW China.

Image: http://apps.kew.org/herbcat/getImage.do?imageBarcode=K000742801

http://ts-den.aluka.org/fsi/img/size1/alukaplant/e/phase_01/e0000/e00301190.jpg

GenBank: Sequences from Renner et al. (2007), Sebastian et al. (2010), and many others, e.g., HM597016, HM597017.

Comments: Based on molecular data, *Cucumis* has about 25 species in Asia and Australia. *Cucumis hystrix* is the closest wild relative of the cucumber, *Cucumis sativus* (Sebastian et al. 2010).

**15. *Cucumis indicus*** Ghebretinsae & Thulin, Novon 17(2): 177. 2007.

*Melothria ritchiei* Chakrav., J. Bombay Nat. Hist. Soc. 50(4): 898, fig. A–K. 1952.

*Cucumella ritchiei* (Chakrav.) C. Jeffrey, Kew Bull. 19: 215. 1965, non *Cucumis ritchiei* (C.B. Clarke) Ghebretinsae & Thulin.

Type: India, Maharasthra, Bombay Presidency, Savantvadi State, Ram Ghat, *D. Ritchie 67* (BM, E; http://plants.jstor.org/specimen/e00187895 ).

Distribution in India: Kerala, Maharashtra (Naithani, 1990). **Endemic.**

Image: http://ts-den.aluka.org/fsi/img/size2/alukaplant/e/phase_01/e0005/e00187895.jpg

GenBank: Sequences from Sebastian et al. (2010), e.g., HM597078, HM596966.

Comments: Molecular phylogenetic data show that the former genus *Dicaelospermum*, with the species *Dicaelospermum ritchiei* C.B. Clarke (1879), is nested inside *Cucumis*. The resulting nomenclatural transfer meant that the epithet “*ritchiei*” is occupied within the genus. A replacement name therefore became necessary with the transfer of *Melothria ritchiei* to *Cucumis*.

**16. *Cucumis javanicus*** (Miq.) Ghebretinsae &Thulin, Novon 17(2). 177. 2007.

*Karivia javanica* Miq., Fl. Ned. Ind. 1: 661. 1855.

*Mukia javanica* (Miq.) C. Jeffrey in Hooker’s Icon. Pl.37: 3, pl. 3661. 1969.

*Melothria javanica* (Miq.) Panigrahi & Misra, J. Econ. Tax. Bot. 5: 416. 1984.

Type: Java, *T. Horsfield*s.n. (BM, K, U).

*Melothria assamica* Chakrav., J. Bombay Nat. Hist. Soc. 50(4): 897.1952.

Type: India, Assam, Cachar, *R. L. Keenan s. n*. (K).

*Melothria assamica* Chakrav. var. *scabra* Chakrav., J. Bombay. Nat. Hist. Soc. 50(4): 898. 1952.

*Melothria javanica* (Miq.) Panigrahi & Misra var. *scabra* (Chakrav.) Naithani, Flowering Plants of India, Nepal & Bhutan 179. 1990.

Type: India, Assam, Goalpara, Chirang Duar, Dec. 1890, *King’s collector*s.n. (CAL, 2 sheets, photos available from SSR).

Distribution in India: Assam.

Distribution outside India: Java, China, and Thailand.

GenBank: Sequences from Renner et al. (2007) and Sebastian et al. (2010), e.g., HM597079, EF174484.

Comment: De Wilde and Duyfjes (2006a) synonymized *Melothria assamica* under *Cucumis javanicus*, which they treated as *Mukia javanica*, a genus that based on molecular data, however, is deeply nested inside *Cucumis*.

**17. *Cucumis leiospermus*** (Wight & Arn.) Ghebretinsae & Thulin, Novon 17(2): 177. 2007.

*Bryonia leiosperma* Wight & Arn., Prodr. Fl. Ind. Orient. 1: 345. 1834.

*Mukia leiosperma* (Wight & Arn.) Arn., Madras J. Lit. Sci. 12: 50. 1840.

*Melothria leiosperma* (Wight & Arn.) Cogn. in A. & C. DC., Monogr. Phan. 3: 622. 1881.

Syntypes: India, Tamil Nadu, Dindygul Hills, *Wallich Cat. no. 6708* (K); Chennai, Palni Hills, *R. Wight 1112* (BR, K). The Wallich specimen was chosen as lectotype by

Jeffrey (1969).

Distribution in India: Andhra Pradesh, Assam, Karnataka, Madhya Pradesh, Maharashtra, Manipur, Rajasthan, Sikkim, Tamil Nadu.

Distribution outside India: Sri Lanka.

Photos by A. Pandey: http://farm8.staticflickr.com/7273/7859393808_2314892118_m.jpg

http://farm9.staticflickr.com/8307/7859413918_cff80f25db_m.jpg

GenBank: Sequences from Sebastian et al. (2010), e.g., HM597080, HM596911.

Comments: An understudied relative of the cucumber and melon.

**18. *Cucumis maderaspatanus*** L., Sp. Pl. 2: 1012. 1753.

*Mukia maderaspatana* (L.) M.Roem., Fam. Nat. Syn. Monogr. 2: 47. 1846

*Melothria maderaspatana* (L.) Cogn. in A. & C. DC., Monogr. Phan. 3: 623. 1881.

Type: India, “Cucumis Maderaspatensis fructu minimo” in Plukenet, Phytographia t. 170. f. 2. 1692. Typotype Herb. Sloane 95: 201 (BM-SL), designated by Meeuse, Bothalia 8: 14. 1962.

*Bryonia cordifolia* L., Sp. Pl. 2: 1012. 1753.

*Coccinia cordifolia* (L.) Cogn. in A. & C. DC., Monogr. Phan. 3: 623. 1881.

Type: “Habitat in Zeylonia,” Lectotype: Herb. Hermann 2: 22, No. 354 (BM-000621582), designated by Jeffrey (1967).

*Bryonia scabrella* L.f., Suppl. Pl. 424. 1782 (“1781”).

*Mukia scabrella* (L.f.) Arn., J. Bot. 3: 276. 1841.

Type: Northwest India, *Royle* s.n. (K, CAL photo available from SSR).

Distribution in India: Andhra Pradesh, Arunachal Pradesh, Bihar, Delhi, Gujarat, Himachal Pradesh, Karnataka, Madhya Pradesh, Maharashtra, Mizoram, Rajasthan, Tamil Nadu, Tripura.

Distribution outside India: Bhutan, China, Myanmar, Nepal, Pakistan, Sri Lanka.

Images: Efloraofindia at https://sites.google.com/site/efloraofindia/species/a---l/cl/cucurbitaceae/mukia/cucumis-maderaspatanus

http://www.flowersofindia.net/catalog/slides/Madras%20Pea%20Pumpkin.html

*Flora of Pakistan*: http://www.tropicos.org/Name/9200868?projectid=32

GenBank: Many sequences from Kocyan et al. (2007), Sebastian et al. (2010), and other studies.

**19. *Cucumis melo*** L., Sp. Pl. 2: 1011. 1753.

Lectotype: Herb. Linn. No. 1152.8 (LINN), designated by Meeuse, Bothalia 8: 61. 1962.

*Bryonia callosa* Rottler, Neue Schriften der Ges. Naturf. Freunde Berlin 4: 210. 1803.

*Cucumis callosus* (Rottler) Cogn. in Engl. Pflanzenr. IV. 275, 2: 129. 1924.

Type: India, Tamil Nadu, Deccan, *Rottler* s.n. (K?). Note: Rottler was a missionary in the Danish Settlement at Tranquebar (150 miles south of Madras) in the years after 1768.

*Cucumis pubescens* Willd., Sp. Pl., ed. 4(1): 614. 1805.

Type: Plant cultivated at Berlin; *C.L. Willdenow*s.n. (B-W, IDC microfiche 7440, specimen number 18048).

*Cucumis momordica* Roxb. Fl. ind. 3: 720. 1832.

Type: India, *W. Roxburgh*s.n. (K?).

*Cucumis trigonus* Roxb., Fl. Ind. 3: 722. 1832.

Lectotype: India, *W. Roxburgh*s.n. (K), designated by Kirkbride, Biosyst. Monogr. *Cucumis* 115. 1993.

*Cucumis melo* var. *pubescens* (Willd.) Kurz, J. Asiat. Soc. Bengal, Pt. 2, Nat. Hist. 46(2): 103. 1877.

*Cucumis melo* var. *culta* Kurz., J. Asiat. Soc. Bengal, Pt. 2, Nat. Hist. 46(2): 102. 1877.

*Cucumis melo* var. *agrestis* Naudin, Ann. Sci. Nat., Bot. sér. 4,11: 73. 1859. Lectotype: India, Union Territory, Puducherry [Pondicherry]: seeds sent by Jules Lépeire (plants cultiv. at Musée d’Histoire Naturelle, Paris); 1859; *Naudin* s.n. (P), designated by J.H. Kirkbride in Biosyst. Monogr. Gen. Cucumis 81. 1993.

*Cucumis melo* ssp. *agrestis* (Naudin) Pangalo in Zhukovsky, La Turquie agricole 534. 1933.

*Cucumis melo* forma *agrestis* (Naudin) W.J.de Wilde & Duyfjes, Sandakania 17: 55. 2008.

Distribution in India: Andhra Pradesh, Assam, Karnataka, Kerala, Madhya Pradesh, Maharashtra, Manipur, Rajasthan, Tamil Nadu, Tripura, Uttar Pradesh.

Distribution outside India: Widely cultivated.

Images: See efloraofindia http://www.flowersofindia.net/catalog/slides/Wild%20Melon.html

Type: http://apps.kew.org/herbcat/getImage.do?imageBarcode=K000634447

http://apps.kew.org/herbcat/getImage.do?imageBarcode=K000794987

http://apps.kew.org/herbcat/getImage.do?imageBarcode=K000742804

GenBank: Numerous sequences from the three plant organellar genomes.

Comments: Sequences representing *Cucumis callosus*, *Cucumis pubescens*, and *Cucumis trigonus* all cluster with *Cucumis melo* (Sebastian et al. 2010) and likely present wild progenitors of domesticated *Cucumis melo*. Jeffrey (1980) preferred to list *Cucumis trigonus* as a separate species, and Chakravarty (1982) mentions two further varieties, *Cucumis melo* var. *momordica* Duthie & Fullar and var. *utilissima* Duthie & Fullar. Without specimens, these varieties cannot be assessed.

**20. *Cucumis prophetarum*** L., Cent. I. Pl. 33. 1755.

Type: Arabia, *D. Hasselquist*. Lectotype: Herb. Linn. No. 1152.4 (LINN), designated by Jeffrey (1962).

Distribution in India: Andhra Pradesh, Goa, Gujarat, Karnataka, Kerala, Madhya Pradesh, Maharashtra, Punjab, Rajasthan, Tamil Nadu.

Distribution outside India: Pakistan to North Africa.

Images: See efloraofindia at https://sites.google.com/site/efloraofindia/species/a---l/cl/cucurbitaceae/cucumis/cucumis-prophetarum

*Flora of Pakistan* http://www.tropicos.org/Name/9200833?projectid=32

GenBank: Sequences from Renner et al. (2007) and Sebastian et al. (2010), e.g., DQ785879, DQ785837.

**21. *Cucumis ritchiei*** (C.B. Clarke) Ghebretinsae & Thulin, Novon 17(2): 178. 2007.

*Dicoelospermum ritchiei* C.B. Clarke, Fl. Brit. India 2: 630. 1879.

*Mukia ritchiei* (C.B. Clarke) W.J.de Wilde & Duyfjes, Thai Forest Bull., Bot. 34: 45. 2006.

Type: India, Karnataka, Bombay Presidency, Belgaum, *D. Ritchie 316* (K).

Distribution in India: Karnataka, Kerala, Maharashtra, Punjab, Tamil Nadu. **Endemic.**

Photos taken at Fort Panhala in Kolhapur District: http://farm9.staticflickr.com/8305/7859345614_e613f0019d_m.jpg

http://farm9.staticflickr.com/8284/7859384216_f591b5418d_m.jpg

GenBank: Sequences from Kocyan et al. (2007) and Sebastian et al. (2010), e.g., DQ536546, HM597095.

Comments: Molecular phylogenetic data show that the former genus *Dicaelospermum* is embedded among the Asian species of *Cucumis*.

**22. *Cucumis sativus*** L., Sp. Pl. 2: 1012. 1753.

Lectotype: Herb. Burser 17: 97 (UPS), designated by ten Pas et al., Taxon 34: 290. f. 1–3. 1985.

*Cucumis sativus* var. *sikkimensis* Hook.f., Bot. Mag. 102: t. 6206. 1876.

Type: Commonly cultivated in the Eastern Himalaya Mountains, 1848; *Hooker* s.n.

*Cucumis hardwickii* Royle, Ill. Bot. Himal. Mts. 220. t. 47. 1835.

Type: Northwestern India, *J.F. Royle*s.n. (LIV).

*Cucumis sativus* L. forma *hardwickii* (Royle) W.J.de Wilde & Duyfjes, Sandakania 17: 58. 2008.

Distribution in India: All evidence points to northern India (Ganges region) as the place where wild cucumbers were first cultivated and where wild populations still occur (Sebastian et al., 2010). Wild cucumbers can be distinguished from cultivated (feral) forms by their extremely bitter fruits.

Distribution outside India: Bhutan, China, Myanmar, Nepal, Thailand.

Image: http://www.flowersofindia.net/catalog/slides/Cucumber.html

GenBank: The genomes of three domesticated lines of cucumber have been sequenced, the American pickling cucumber, a Polish line, and a Chinese line (Huang et al. 2009).

Comments: The wild progenitors of domesticated cucumber still occur in India (Sebastian et al. 2010).

**23. *Cucumis setosus*** Cogn. in A. & C. DC., Monogr. Phan. 3: 491. 1881.

Type: India, Karnataka, Western Ghats, Belgaum, *Ritchie 321* (E, K).

Distribution in India: Gujarat, Karnataka, Madhya Pradesh, Maharashtra, Rajasthan. **Endemic.**

Photos by Suresh Jagtap, taken near Purandhar fort:

http://farm9.staticflickr.com/8443/7859357598_fd99ecd49b_m.jpg

http://farm9.staticflickr.com/8304/7859369892_28668e0fd2_m.jpg

GenBank: Sequences from Sebastian et al. (2010), e.g., HM597106, HM596985.

Comments: A distinct species.

**24. *Cucumis silentvalleyi*** (Manilal, T. Sabu & P. J. Mathew) Ghebretinsae & Thulin, Novon 17: 178. 2007.

*Cucumella silentvalleyi* Manilal, T. Sabu & P. J. Mathew, Acta Bot. Indica 13: 283. 1985. (as *silentvalleyii*)

Type: India, Kerala, Palghat Distr., Silent Valley, Poochapara, alt. 1370 m, 20 Oct. 1982, *T. Sabu SV10662* (K, MH not seen).

Distribution in India: Kerala. **Endemic**.

Image: Photos taken near the type locality by Natalia Filipowicz, available from SSR.

GenBank: Sequences from Sebastian et al. (2010), e.g., HM597038, HM596931.

Comments: This species is one of c. 25 Asian and Australian species of *Cucumis* (Sebastian et al. 2010).

**25. *Cucurbita argyrosperma*** C.Huber, Cat. Graines 8. 1867.

Type: A cultivated plant.

*Cucurbita mixta* Pangalo, Bull. Applied Bot., Leningrad 1929-30, 23(3): 264. 1930.

Type: Mexico, Guatemala.

Distribution in India: Cultivated?

Distribution outside India: Native to Mesoamerica, widely cultivated.

Image: Many images can be found online of plants grown outside India.

GenBank: Many sequences from Sanjur et al. (2002) and further studies.

Comment: Jeffrey (1980) included this species (as *Cucurbita mixta*) in his checklist of Indian Cucurbitaceae, but it is unclear to what extent it is cultivated in India today.

**26. *Cucurbita ficifolia*** Bouché, Verh. Vereins Beford. Gartenbaues Königl. Preuss. Staaten 12: 205. 1837.

Type: So far unknown.

Distribution in India: Meghalaya (Naithani, 1990). Cultivated.

Distribution outside India: Native to Mesoamerica or northern South America, widely cultivated.

Image: See efloraofindia at https://sites.google.com/site/efloraofindia/species/a---l/cl/cucurbitaceae/cucurbita/cucurbita-filicifolia

GenBank: Sequences from Sanjur et al. (2002) and Kocyan et al. (2007), e.g., HQ438599, DQ536665.

Comments: *Cucurbita* has about 15 wild species in tropical and subtropical America (M. Nee, New York Botanical Garden, pers. comm., Feb. 2010) and five domesticated ones cultivated worldwide (*Cucurbita argyrosperma*, *Cucurbita ficifolia*, *Cucurbita maxima*, *Cucurbita moschata*, and *Cucurbita pepo*).

**27.** ***Cucurbita maxima*** Duchesne, Essai Hist. Nat. Courges 7, 12. 1786.

Type: From a cultivated plant (not found); neotype: Melo-pepo fructa albo Tournefort Inst. 1: 106. T. 34 1700.

*Cucurbita maxima* var. *badagarensis* Mudaliar, J. Bombay Nat. Hist. Soc. 49: 242. 1950.

Type: India, Malbar District, cultivated, collector unknown, Madras Herbarium No. 93177 and 93178 (MH).

Distribution in India: Andhra Pradesh, Arunachal Pradesh, Assam, Bihar, Goa, Gujarat, Himachal Pradesh, Karnataka, Kerala (Naithani, 1990), Madhya Pradesh, Maharashtra, Rajasthan, Tamil Nadu, Tripura, Uttar Pradesh, Uttarakhand.

Distribution outside India: Native to Central America.

Image: efloraofindia at https://sites.google.com/site/efloraofindia/species/a---l/cl/cucurbitaceae/cucurbita/cucurbita-maxima

GenBank: Numerous sequences from the three plant organellar genomes.

Comments: Winter squash is cultivated throughout India.

**28. *Cucurbita moschata*** (Duchesne ex Lam.) Duchesne, Essai Hist. Nat. Courges 7. 1786.

*Cucurbita pepo* var. *moschata* Duchesne ex Lam., Encycl. 2: 152. 1786.

Type: “M. Duchesne presume que cette gourge est la meme que le cucurbita major rotunda, flore luteo, folia aspero de G.B. Pin 312 qui est le Cucurbita India rotunda de Dalechampe (Lugd. 616).”

Distribution in India: Arunachal Pradesh, Assam, Bihar, Delhi, Goa, Himachal Pradesh, Kerala, Madhya Pradesh, Maharashtra, Manipur, Mizoram, Rajasthan, Sikkim, Tamil Nadu, Tripura, Uttar Pradesh.

Distribution outside India: Native to Central or South America.

Image: See efloraofindia at https://sites.google.com/site/efloraofindia/species/a---l/cl/cucurbitaceae/cucurbita/cucurbita-moschata

GenBank: Numerous sequences from the three plant organellar genomes.

**29. *Cucurbita pepo*** L., Sp. Pl. 2: 1010. 1753.

Lectotype: Herb. Linn. No. 1151.4 (LINN), designated by Keraudren-Aymonin in Aubréville & Leroy (ed.), Fl. Cambodge Laos Viêt-Nam 15: 105. 1975.

Distribution in India: Arunachal Pradesh, Assam, Bihar, Delhi, Goa, Himachal Pradesh, Karnataka, Kerala, Madhya Pradesh, Maharashtra, Manipur, Mizoram, Punjab, Tamil Nadu, Tripura, Uttar Pradesh. Cultivated.

Distribution outside India: Native to Central or South America.

Image: http://www.flowersofindia.net/catalog/slides/Pumpkin.html

GenBank: Numerous sequences from the three plant organellar genomes.

Comments: See Barrie (Taxon 55: 795-796. 2006) for a history of this name. Chakravarty (1982) also mentions the varietis var. *melopepo* Alef. and var. *ovigera* Alef.; we are unsure about their validity.

**30. *Cyclanthera pedata*** (L.) Schrad., Index Seminum, Gottingen 1831: 2. 1831; emend in Linnaea 8(Litt.): 22–27. 1833.

*Momordica pedata* L., Sp. Pl. 2: 1009. 1753.

Lectotype: Peru, “Momordica fructu striato, Laevi, vulgo Caigua” in Feuillée, J. Obs., 2: 754. t. 41. 1714, designated by Jeffrey in Kew Bull. 34: 796. 1980.

Distribution in India: Cultivated in northern India.

Distribution outside India: Native to South America; cultivated also in Bhuthan.

Images: See efloraofindia at https://sites.google.com/site/efloraofindia/species/a---l/cl/cucurbitaceae/cyclanthera/cyclanthera-pedata

GenBank: Sequences from Decker-Walters et al. (2004), e.g., AY396221, AJ748597.

Comments: *Cyclanthera* has c. 40 species in the Southwestern USA, Mexico, Central and South America, one species on the Galapagos archipelago (Schaefer and Renner 2011a).

**31. *Dactyliandra welwitschii*** Hook.f., Fl. Trop. Afr. 2: 557. 1871.

*Ctenolepis welwitschii* (Hook.f.) Jafri, Fl. Karachi 327 (1966)

Type: Lower Guinea, sandy thickets in Luanda, *Welwitsch 832* (BM).

Distribution in India: Gujarat, Haryana, Rajasthan (fide Chakravarty, 1982).

Distribution outside India: Southwest Africa (Namibia, Angola); coastal West Pakistan (Karachi; Khatoon, 2006).

Image: Nothing reliable found online.

GenBank: Sequences from Kocyan et al. (2007) and Schaefer and Renner (2011b), e.g., HQ201973, DQ535750.

Comments: The genus *Dactyliandra* has two African species of which one, *Dactyliandra welwitschii*, also occurs in India and Pakistan (Bhandari and Singh 1964; Khatoon 2006), apparently as a natural introduction since the species has no known uses and is not cultivated.

**32. *Diplocyclos palmatus*** (L.) C. Jeffrey, Kew Bull. 15(3): 325. 1962.

*Bryonia palmata* L., Sp. Pl. 2: 1012. 1753, excl. syn.

*Coccinia palmata* M.Roem. Synopsis peponiferarum 93. 1846.

Lectotype: Sri Lanka, Herb. Hermann 2: 58, No. 353 (BM-000621700), designated by Jeffrey (1962).

*Diplocyclos palmatus* var. *walkeri* (Chakrav.) Babu, Herb. Fl. Dehra Dun 198. 1977.

*Bryonopsis laciniosa* (L.) Naudin var. *walkeri* Chakrav., Bot. Surv. India 17(1): 183 (1959).

Type: Sri Lanka, *Walker* s.n. (E).

Distribution in India: Andhra Pradesh, Arunachal Pradesh, Bihar, Jharkhand, Goa, Gujarat, Himachal Pradesh, Jammu & Kashmir, Karnataka, Kerala, Madhya Pradesh, Chhattisgarh, Maharashtra, Manipur, Rajasthan (Naithani, 1990), Tamil Nadu, Tripura, Uttar Pradesh.

Distribution outside India: Bhutan, China, Nepal, Pakistan, Thailand, South Japan, Sri Lanka, Philippines, Indonesia, Peninsular Malaysia, Papua New Guinea, NE Australia.

Images: See efloraofindia at https://sites.google.com/site/efloraofindia/species/a---l/cl/cucurbitaceae/diplocyclos/diplocyclos-palmatus

Flora of Pakistan: http://www.tropicos.org/Name/9201644?projectid=32

GenBank: Sequences from Kocyan et al. (2007) and Holstein and Renner (2011), e.g., DQ536671, DQ536769.

Comments: The other three species of *Diplocyclos* occur in tropical Africa. The name *Bryonopsis laciniosa* (L.) Naudin refers to a species that does not occur in India (see *Misapplied names and species erroneously or doubtfully recorded from India*).

**33. *Gomphogyne cissiformis*** Griff., Account Bot. Coll. Cantor 26, pl. 4: 1–7. 1845. emend, J. Asiat. Soc. Bengal 23(7): 645. 1854.

Type: Himalaya Range, *Edgeworth* 88 (neotype K), designated by Keraudren-Aymonin (1975)

*Gomphogyne cissiformis*var. *villosa* Cogn in A. & C. DC., Monogr. Phan. 3: 925. 1881.

*Gomphogyne cissiformis* forma *villosa* (Cogn.) Mizush., J. Jap. Bot. 41: 259. 1966.

Type: India, Sikkim, *Hooker* s.n., 2 Oct. 1843 (K).

Distribution in India: Arunachal Pradesh, Himachal Pradesh, Mizoram, Sikkim, Uttar Pradesh, West Bengal.

Distribution outside India: Nepal, Bhutan, China (Yunnan).

Image: Nothing reliable found online.

GenBank: Sequences from Schaefer et al. (2009), e.g., EU436354.

Comments: The genus *Gomphogyne* has at least two species, *Gomphogyne cissiformis* Griff. and *Gomphogyne nepalensis* W.J.de Wilde & Duyfjes (De Wilde et al. 2007). A third species, *Gomphogyne cirromitrata* W.J.de Wilde & Duyfjes, based on molecular data, belongs in *Hemsleya* (as *Hemsleya cirromitrata* (W.J.de Wilde & Duyfjes) H. Schaef. & S.S. Renner; Schaefer and Renner 2011b).

**34.** ***Gynostemma pentaphyllum*** (Thunb.) Makino, Bot. Mag. (Tokyo) 16: 179. 1902.

*Vitis pentaphylla* Thunb., Syst. Veg., ed. 14: 244. 1784.

Type: Japan, *Thunberg 5858* (UPS).

*Gynostemma pedatum* Blume, Bijdr. Fl. Ned. Ind. 1: 23. 1825 (as *pedata*).

Lectotype: Java, Tjanjor & Krawang, *Blume 1429* (L, barcode L0588327), designated by De Wilde and Duyfjes, Blumea 52(2): 271. 2007.

*Gynostemma simplicifolium* Blume, Bijdr. Fl. Ned. Ind. 1: 24. 1825 (as *simplicifolia*).

*Gynostemma pentaphyllum* forma *simplicifolium* (Blume) W.J.de Wilde & Duyfjes, Blumea 52(2): 271. 2007.

Lectotype: Java, Mt Krawang, *Blume 1493* (L, barcode L0588361), designated by De Wilde and Duyfjes, Blumea 52(2): 271. 2007.

*Gynostemma laxum* (Wall.) Cogn. in A. & C. DC., Monogr. Phan. 3: 914. 1881 (as *laxa*).

*Zanonia laxa* Wall., Pl. Asiat. Rar. 2: 29. 1831.

Type: Bangladesh [India], Silhet; *Wallich Cat. 3727 A-B* (K, K-W, BM).

(Further synonyms are listed in De Wilde and Duyfjes 2007.)

Distribution in India: Cultivated in Arunachal Pradesh, Assam, Himachal Pradesh, Manipur, Meghalaya, Mizoram, Nagaland, Sikkim, Tamil Nadu, Tripura, Uttar Pradesh, West Bengal.

Distribution outside India: Bangladesh, Bhutan, China, Myanmar, Sri Lanka.

Image: Many images of this frequently cultivated species are found online.

GenBank: Sequences from Zhang et al. (2006), Chen et al. (2010) and other studies.

Comments: The species is used to make herbal teas. Its natural range is currently unclear. The genus *Gynostemma* has some ten species, all in Asia (Schaefer and Renner 2011a).

**35. *Hemsleya macrocarpa*** (Cogn.) C. Y. Wu ex C. Jeffrey, Kew Bull. 36: 739. 1982.

*Gomphogyne macrocarpa* Cogn. in Engl. Pflanzenr. IV. 275, 1 (Heft 66): 40. 1916.

Holotype: India, Manipur, Laimatak, alt. 1300 m, November 1907, *A. Meebold 6522* (Wroclaw University, Poland: BRSL, not seen).

Distribution in India: Assam, Arunachal Pradesh, Manipur, Nagaland (Naithani, 1990).

Distribution outside India: China (Yunnan).

Image: Nothing reliable found online.

GenBank: Sequences from Li et al. (2010) and Li et al. (2011), e.g., JF976573, JN044854.

Comments: *Hemsleya* is thought to comprise 30 species mostly in China, a few in Indochina and East Malesia (Schaefer and Renner 2011a). Further synonyms of *Hemsleya macrocarpa* are listed in Lu et al. (2011).

**36.** ***Herpetospermum darjeelingense*** (C.B. Clarke) H.Schaef. & S.S.Renner, Taxon 60(1): 134. 2011.

*Edgaria darjeelingensis* C.B.Clarke, J. Linn. Soc. 15: 114. 1876.

Type: India, West Bengal, Darjeeling, 1 Oct 1875, *C.B. Clarke 26857* (CAL photo available from SSR, K).

*Edgaria darjeelingensis* var. *clarkeana* S. N. Biswas, J. Econ. Taxon. Bot. 18(1): 173, f. A-1-6. 1994 (as *clarkiana*).

Type: India, West Bengal, Darjeeling, alt. 2100 m, 9 Sep. 1875, *Griffith* s.n. (K).

Distribution in India: Arunachal Pradesh, Sikkim, West Bengal.

Distribution outside India: Bhutan, Nepal, China (Xizang).

Image: See above, photo of type collection.

GenBank: Sequences from Kocyan et al. (2007), e.g., DQ536550.

Comments: The genus *Herpetospermum* has three species in India, Myanmar, Nepal, Tibet, and China (Yunnan). In the herbarium, *Herpetospermum darjeelingense* can be confused with *Herpetospermum pedunculosum*, from which it is distinguished by its narrow and glabrous calyx-tube with filiform lobes (Chakravarty, 1982).

**37. *Herpetospermum pedunculosum*** (Ser.) Baill. Hist. Pl. 8:445. 1885.

*Bryonia pedunculosa* Ser., Prodr. 3: 306. 1828.

Isotypes: Nepal, *Wallich* s.n. (G-DC, K), *Wallich 6761* (K-W).

Distribution in India: Arunachal Pradesh, Assam, Himachal Pradesh, Manipur, Meghalaya, Nagaland, Sikkim, Uttar Pradesh, West Bengal.

Distribution outside India: Bhutan, Nepal, China.

Images: efloraofindia at https://sites.google.com/site/efloraofindia/species/a---l/cl/cucurbitaceae/herpetospermum/herpetospermum-pedunculosum

http://www.flowersofindia.net/catalog/slides/Beej%20Karela.html

GenBank: Sequences from Schaefer and Renner (2011) and Li et al. (2011), e.g., JN044888, JF941910.

Comments: The *Flora of British India* (Clarke, 1879) lists the name *Herpetospermum caudigerum* Wall. ex C.B. Clarke, but that is an illegitimate name for *Bryonia pedunculosa* Ser.

**38. *Herpetospermum tonglense*** (C.B. Clarke) H. Schaef. & S.S. Renner, Taxon 60(2): 615 (2011c).

*Warea tonglensis* C.B. Clarke in J. Linn. Soc., Bot. 15: 129. 1876.

*Biswarea tonglensis* (C.B. Clarke) Cogn. in A. & C. DC., Monogr. Phan. 3: 403. 1881.

Type: India, West Bengal, Darjeeling, Rungbee, *C.B. Clarke 12183A* (K).

Distribution in India: Assam, Manipur, Sikkim, West Bengal, Eastern Himalyan ranges

Distribution outside India: China, Nepal, Myanmar.

Image: Nothing reliable found online.

GenBank: Sequences from Kocyan et al. (2007), e.g., JQ933236, DQ536637.

Comments: Based on plastid and nuclear gene topologies, this species is the sister species to *Herpetospermum pedunculosum*, and Schaefer and Renner (2011a, b) therefore merged the monotypic genus *Biswarea* with *Herpetospermum*.

**39. *Hodgsonia heteroclita*** (Roxb.) Hook.f. & Thomson, Proc. Linn. Soc. London 2: 257. 1854 (“1855”).

*Trichosanthes heteroclita* Roxb., Fl. Ind. 3: 705-707. 1832.

Type: Bangladesh [India, Bengal] *Hodgsonia Roxburgh* s.n. (K) “Native of the eastern parts of Bengal. From Silhet Mr. Robert Keith Dick, the Judge of that district, sent plants to the botanic garden in 1805.”

Distribution in India: Arunchal Pradesh, Assam, Meghalaya, Sikkim, Tripura, West Bengal.

Distribution outside India: Bangladesh, Bhutan, Cambodia, Laos, Myanmar, Thailand, Vietnam.

Image: See efloraofindia at https://sites.google.com/site/efloraofindia/species/a---l/cl/cucurbitaceae/hodgsonia/hodgsonia-macrocarpa

GenBank: Sequences from Schaefer and Renner (2011b) and De Boer et al. (2012), e.g., HE661403, HQ201981.

Comment: Jeffrey (1980) considered *Hodgsonia heteroclita* a synonym of *Hodgsonia macrocarpa* (Blume) Cogn. (see under misapplied names and species erroneously or doubtfully recorded from India), while De Wilde and Duyfjes (2001) recognize two species.

**40. *Indofevillea khasiana*** Chatterjee, Kew Bull. 2(2): 121. f.1-7. 1948 (“1947”).

Type: India, Meghalaya [Assam], Khasia Hills, 1886, *G.Mann*s.n. (CAL, 2 sheets, photos available from SSR).

Distribution in India: Arunachal Pradesh, Assam, Meghalaya.

Distribution outside India: Bhutan, Tibet.

Image: See efloraofindia at https://sites.google.com/site/efloraofindia/species/a---l/cl/cucurbitaceae/indofevillea-khasiana/indofevillea-khasiana

GenBank: Sequences from Schaefer and Renner (2011b), e.g., DQ501256, HQ201983.

Comments: Based on molecular data, *Indofevillea khasiana* represents an isolated ancient lineage of Cucurbitaceae (Schaefer and Renner 2011a; our Fig. 1).

**41. *Kedrostis courtallensis*** (Arn.) C. Jeffrey, Kew Bull. 15: 353. 1962.

*Bryonopsis courtallensis* Arn., J. Bot. 3: 274. 1841.

Type: Sri Lanka, *Wight 1147* (K).

*Cerasiocarpum zeylanicum* (Thwaites) C.B. Clarke, Fl. Brit. India 2: 629. 1879.

*Aechmandra zeylanica* Thwaites, Enum. Pl. Zeyl. 2: 125. 1859.

Type: Sri Lanka, *Thwaites 3002* (CAL, 2 sheets, photos available from SSR), *3500* (CAL, 2 sheets, K).

*Cerasiocarpum bennettii* (Miq.) Cogn. in A. & C. DC., Monogr. Phan. 3: 729. 1881.

*Kedrostis bennettii* (Miq.) W.J.de Wilde & Duyfjes, Reinwardtia 12(2): 130. 2004.

*Bryonopsis bennettii* Miq., Fl. Ned. Ind. 1: 657. 1855.

Type: Java, in Banjoemas door, *T. Horsfield*s.n. (K, U).

Distribution in India: Andhra Pradesh, Karnataka, Kerala, Maharashtra, Tamil Nadu.

Distribution outside India: Myanmar, Sri Lanka.

Image: Nothing reliable found online.

GenBank: No published sequences available.

Comments: The genus *Kedrostis* comprises about 20 species in tropical and subtropical Africa and Arabia, six species in Madagascar, and perhaps four in India, Sri Lanka, and West Malesia (De Wilde and Duyfjes 2004a; Schaefer and Renner 2011a).

**42. *Kedrostis foetidissima*** (Jacq.) Cogn. in A. & C. DC., Monogr. Phan. 3: 634. 1881.

*Trichosanthes foetidissima* Jacq., Collectanea 2: 841. 1788.

Type: West Africa, plant cultivated in Vienna and depicted in Jacq. , Collectanea 4. 1790, pl. 624.

*Bryonia rostrata* Rottler, Neue Schriften der Ges. Naturf. Freunde Berlin 4: 212. 1803.

*Aechmandra rostrata* (Rottler) Arn., J. Bot. 3: 274. 1841.

*Rynchocarpa rostrata* (Rottler) Naudin, Ann. Sci. Nat., Bot. sér. 4,16: 177. 1862.

*Kedrostis rostrata* (Rottler) Cogn. in A. & C. DC., Monogr. Phan. 3: 636. 1881. Type: India, Tamil Nadu, Nandaradah, *Rottler 766* (B-W, K).

Distribution in India: Andhra Pradesh, Gujarat, Karnataka, Madhya Pradesh, Maharashtra, Tamil Nadu. Cultivated.

Distribution outside India: A West African species cultivated in India, Bangladesh, Myanmar, Pakistan, Sri Lanka.

Image: For a detailed description and links to images see http://plants.jstor.org/flora/ftea001850

GenBank: Sequences from a plant from Benin (Africa): AM981179, AM981180.

Comments: Fruits and leaves are used as a vegetable, and the roots (and fruits) also medicinally.

**43. *Lagenaria siceraria*** (Molina) Standl., Publ. Field Mus. Nat. Hist. Chicago, Bot. Ser. 3: 435. 1930.

*Cucurbita siceraria* Molina, Sag. Stor. Nat. Chili 133. 1782.

Type: Chile, *Molina* s.n. (lost), lectotype: LINN-1151.1

*Cucurbita lagenaria* L., Sp. Pl. 2: 1010. 1753.

Type: America, Herb. Linn. No. 1151.1 (LINN), designated by Jeffrey (1967).

Distribution in India: Cultivated throughout India.

Distribution outside India: Native of tropical Africa.

Image: See efloraofindia at https://sites.google.com/site/efloraofindia/species/a---l/cl/cucurbitaceae/lagenaria/lagenaria-siceraria and http://www.flowersofindia.net/catalog/slides/Bottle%20Gourd.html

GenBank: Hundreds of sequences from the three plant organellar genomes.

Comments: The bottle gourd is a native of tropical Africa and is cultivated throughout the tropics (further information and references see Schaefer and Renner 2011a).

**44. *Luffa acutangula*** (L.) Roxb. Hort. Beng. 70. 1814.

*Cucumis acutangulus* L., Sp. Pl. 2: 1011. 1753.

Type: “Habitat in Tataria, China.” Type not designated.

*Luffa amara* Roxb., Fl. Ind. 3: 715. 1832.

*Luffa acutangula* var. *amara* (Roxb.) C.B. Clarke, Fl. Brit. India 2: 615. 1879.

*Luffa acutangula* forma *amara* (Roxb.) W.J.de Wilde & Duyfjes, Sandakania 17: 68. 2008.

Lectotype: India, Ic. Roxb. 460 (K) designated by Jeffrey (1980).

*Luffa hermaphrodita* Singh & Bhandari, Baileya 11(4): 136, Fig. 13. 1964.

Type: India, Rajasthan, cultivated at Botanical Gardens, Jaswant College, Jodhpur from seeds collected at Agra by D. Singh, 20 Aug. 1962, *Bhandari 1527A* (CAL photo available from SSR).

*Cucurbita umbellata* Willd., Sp. Pl., ed. 4(1): 608. 1805.

*Luffa umbellata* (Willd.) M.Roem., Fam. Nat. Syn. Monogr. 2: 63. 1846.

Syntypes: East India, *Klein* 769 (B-W 18033) and *Klein* s.n. (K) fide Jeffrey (1992).

*Luffa kleinii* Wight & Arn., Prodr. Fl. Ind. Orient. 1: 344. 1834.

Type: India, Kreala, Travancore, Mirittupadu, *Klein* s.n. (K?).

Distribution in India: Native and cultivated throughout India.

Distribution outside India: Cultivated worldwide.

Image: See efloraofindia at https://sites.google.com/site/efloraofindia/species/a---l/cl/cucurbitaceae/luffa/luffa-acutangula

http://www.flickr.com/photos/83425416@N02/7649353846/in/photostream

GenBank: Sequences from Kocyan et al. (2007), e.g., HE661305, HE661476.

Comments: The genus *Luffa* has eight species, three in the Neotropics, one in Australia, and four in Africa and Asia. The Indian species are discussed in Pandey et al. (2006). Jeffrey (1980) and later authors treated Herb. Linn. No. 1152/7 (LINN) as the (lecto)type. However, this collection lacks the relevant *Species Plantarum* number and was a post-1753 addition to the herbarium (Jarvis, 2007).

**45. *Luffa cylindrica*** (L.) M.Roem., Fam. Nat. Syn. Monogr. 2: 63. 1846.

*Momordica cylindrica* L., Sp. Pl. 2: 1009. 1753.

Type: Sri Lanka and China. Lectotype: Herb. Linn. No. 1150.9 (LINN), designated by Wunderlin in Ann. Missouri Bot. Gard. 65: 329. 1978.

*Luffa aegyptiaca* Mill., Gard. Dict., ed. 8. Luffa no. 1. 1768.

Type: Presumably a cultivated plant (Jeffrey, 1962). Lectotype: Pepo indicus reticulatus eminibus nigris Herm., Hort. Acad. Lugd.-Bat. Cat.: 482 (1687), designated by Jeffrey (1992).

*Luffa sylvestris* Miq., Fl. Ned. Ind. 1: 666. 1855.

*Luffa cylindrica* (L.) M.Roem. var. *minor* Chakrav., nom. nud. (CAL photo available from SSR).

*Luffa aegyptiaca* forma *sylvestris* (Miq.) W.J.de Wilde & Duyfjes, Sandakania 17: 70. 2008.

Type: “Petola silvestris” in Rumph., Herb. Amboin. 5, p. 409, t. 150. 1746.

Distribution in India: Native and cultivated throughout India.

Distribution outside India: From India to Egypt and Sudan; cultivated widely.

Image: http://www.flowersofindia.net/catalog/slides/Sponge%20Gourd.html

GenBank: Sequences from Sebastian et al. (2012) and numerous other sequences from unvouchered material, some under *Luffa cylindrica*, others under *Luffa aegyptiaca*.

Comments: There has been considerable discussion on whether the correct name for this species is *Luffa cylindrica* or *Luffa aegyptiaca*. The former view was held by Jeffrey (1980), while the latter was adopted by Schubert (Taxon, 24: 174, 1975) and Heiser and Schilling (Biotropica 20(3): 185-191, 1988). Nicolson and colleagues (1988) discuss the issue and prefer *Luffa aegyptiaca*.

**46. *Luffa echinata*** Roxb., Fl. Ind. 3: 716. 1832.

Lectotype: India, Coromandel, Ic. Roxb. 1694 (K), designated by Jeffrey (1980).

*Luffa echinata* var. *longistyla* C.B. Clarke, Fl. Brit. India 2: 615. 1879.

Type: India, *M.P. Edgeworth 3018* (K).

Distribution in India: Assam, Bihar, Gujarat, Himachal Pradesh, Madhya Pradesh, Maharashtra, Tamil Nadu, Uttar Pradesh, Uttarakhand, West Bengal.

Distribution outside India: Wild from Egypt to Niger and maybe further to the West (H. Schaefer, pers. comm., Dec. 2012).

Images: efloraofindia at https://sites.google.com/site/efloraofindia/species/a---l/cl/cucurbitaceae/luffa/luffa-echinata

http://www.flickr.com/photos/83425416@N02/7648878220/in/photostream/

http://www.flickr.com/photos/83425416@N02/7649327834/in/photostream

http://www.flickr.com/photos/83425416@N02/7649413904/in/photostream

GenBank: Sequences from Decker-Walters et al. (2004) and Schaefer et al. (2009), e.g., HE661478, EU436357.

**47. *Luffa graveolens*** Roxb., Fl. Ind. 3: 716. 1832.

Type: Jharkhand (earlier a part of Bihar State) “A native of the Rajmahl hills, from thence the seeds were brought to the botanical garden, where the plants blossom during the rainy season, and the seed ripens about three mounts afterwards.” Lectotype: Ic. Roxb. 1693 (K), designated by Jeffrey (1980).

Distribution in India: Bihar, Maharashtra, Sikkim, Uttar Pradesh.

Distribution outside India: Nepal.

Image: Photos available upon request from A. Pandey or SSR.

GenBank: Sequences from Decker-Walters et al. (2004) and Schaefer et al. (2009), e.g., HE661308, EU436358.

Comments: The application of this name to Australian material was erroneous (Telford et al. 2011). The flowers of *Luffa graveolens* are yellow, while those of *Luffa echinata* are white.

**48. *Momordica balsamina*** L., Sp. Pl. 2: 1009. 1753.

Type: “Habitat in India,” plant cultivated at Hartekamp, The Netherlands. Lectotype: Herb. Linn. No. 1150.1 (LINN), designated by Meeuse in Bothalia 8: 49. 1962.

Distribution in India: Cultivated in Gujarat, Haryana, Rajasthan?

Distribution outside India: Native in the dry savannas of Southernmost Africa and the northern margin of the tropical belt (H. Schaefer, pers. comm., Dec. 2012). Naturalized in parts of tropical Asia, the Americas and most of the Pacific islands.

Image: Flora of Pakistan: http://www.mobot.org/mobot/PakistanImages/154-Cucurbitaceae/Momordica_balsamina.jpg

GenBank: Sequences from Schaefer and Renner (2010), e.g., HM367595, GQ163349.

Comments: *Momordica* has about 60 species in tropical and subtropical Africa, Arabia, (sub) tropical Asia, Malesia and Northeastern Australia (Schaefer and Renner, 2010, 2011a).

**49. *Momordica charantia*** L., Sp. Pl. 2: 1009. 1753.

Type: “Habitat in India.” Lectotype: Herb. Clifford: 451, Momordica 2 (BM-000647445), designated by Jeffrey (1967).

*Momordica charantia* L. var. *muricata* (Willd.) Chakrav., Fasc. Fl. India 11: 92. 1982. *Momordica muricata* Willd., Sp. Pl., ed. 4(1): 602. 1805.

Type: “Habitat in India Orientali,” Plate 10 in Rheede Hort. Mal. Ind. 8. 1688.

Distribution in India: Large fruited forms cultivated all over India as vegetable; small wild forms occur in forest pockets in the Western and Eastern Ghats, Chhattisgarh (Bastar), Jharkhand and all over Central and South India (Joseph and Antony 2010).

Distribution outside India: Native in tropical and subtropical Africa, naturalized in parts of tropical Asia.

Image: efloraofindia at https://sites.google.com/site/efloraofindia/species/a---l/cl/cucurbitaceae/momordica/momordica-charantia

http://www.flowersofindia.net/catalog/slides/Bitter%20Gourd.html

GenBank: Sequences from Schaefer and Renner (2010) and Liao et al. (2012), e.g., DQ501269, HE585488.

**50. *Momordica cochinchinensis*** (Lour.) Spreng., Syst. Veg., ed. 16, 3: 14. 1826. *Muricia cochinchinensis* Lour., Fl. Cochinch. 2: 596. 1790.

Type: Vietnam, *Loureiro* s.n. (BM, http://plants.jstor.org/specimen/bm000944651 ).

*Momordica macrophylla* Gage, Rec. Bot. Surv. India 3: 61. 1908.

Type: Myanmar (Burma), Mergui, April 1911, *A. Meebold*s.n. (CAL?).

Distribution in India: Andaman & Nicobar Islands, Arunachal Pradesh, Assam, Bihar, Karnataka, Manipur, Nagaland, Rajasthan, Tamil Nadu, Tripura, Uttar Pradesh, West Bengal.

Distribution outside India: Native from India in the West to New Guinea/Australia in the Southeast.

Image: efloraofindia at https://sites.google.com/site/efloraofindia/species/a---l/cl/cucurbitaceae/momordica/momordica-cochinchinensis and

http://www.flowersofindia.net/catalog/slides/Chinese%20Cucumber.html

GenBank: Sequences from Schaefer and Renner (2010), e.g., GQ163379, GQ163256.

Comments: Jeffrey (1980; 2001) and De Wilde and Duyfjes (2002) have synonymized *Momordica macrophylla* under *Momordica cochinchinensis*.

**51. *Momordica cymbalaria*** Fenzl ex Naudin, Ann. Sci. Nat., Bot., Sér. 4, 12: 134. 1859.

Type: Africa, Sudan, Kordofan, Mt. Arasch Cool (Arashkol), 9 Oct. 1839, *Kotschy 147* (CAL, 2 sheets, photos available from SSR).

*Momordica tuberosa* (Roxb.) Cogn. in A. & C. DC., Monogr. Phan. 3: 454. 1881, nom. illeg., non Dennst. 1818.

*Luffa tuberosa* Roxb., Fl. Ind. 3: 717. 1832.

Lectotype: India, Ic. Roxb. 461 (K), designated by Jeffrey (1980).

Distribution in India: Andhra Pradesh, Karnataka, Madhya Pradesh, Maharashtra, and Tamil Nadu (fide Parvathi and Kumar 2002).

Distribution outside India: North and East Africa.

Image: See http://en.wikipedia.org/wiki/Momordica_cymbalaria#cite_note-dist-1 .

GenBank: An ITS sequence from an Indian specimen, Karuppusamy 28631 from Andhra Pradesh (Ali et al. 2009; GQ183046), is available and is identical to sequences from Africa (Schaefer and Renner, 2010).

Comments: We disagree with John and Antony (2010) that Jeffrey’s (1980) synonymization of *Luffa tuberosa* with the African *Momordica cymbalaria* is erroneous. Likely introduced to Asia as a vegetable and medicinal plant (Lokesha and Vasudeva 2001).

**52. *Momordica denudata*** (Thwaites) C.B. Clarke, Fl. Brit. India 2: 618. 1879.

*Momordica dioica* Roxb. ex Willd. var. *denudata* Thwaites, Enum. Pl. Zeyl. 2: 126. 1859.

Type: Sri Lanka, *Thwaites 1615* (K, CAL photo available from SSR, PDA).

Distribution in India: Gujarat, Maharashtra, Karnataka, Kerala (Chakravarty 1982).

Distribution outside India: Sri Lanka.

Image: Several of the type specimens can be found online.

GenBank: Schaefer and Renner (2010) generated sequences from *Thwaites 28* (K), collected in Sri Lanka, e.g., GQ163385, GQ163262.

Comments: Joseph and Antony (2010) doubt that Chakravarty (1982) is correct in considering *Momordica denudata* distinct from *Momordica dioica*, while De Wilde and Duyfjes (2002) also consider *Momordica denudata* as distinct.

**53. *Momordica dioica*** Roxb. ex Willd., Sp. Pl., ed. 4(1): 605. 1805.

Type: East India; *Klein 768* (B-Willdenow 18027).

Distribution in India: Joseph and Antony (2010) consider *Momordica dioica* sensu stricto restricted to the Deccan plateau and Central India.

Distribution outside India: Bangladesh, China, Myanmar, Nepal, Pakistan.

Image: efloraofinda at https://sites.google.com/site/efloraofindia/species/a---l/cl/cucurbitaceae/momordica/momordica-dioica also Flora of Pakistan.

GenBank: Sequences from Schaefer and Renner (2010), e.g., GQ163389, GQ163387.

**54. *Momordica sahyadrica*** Kattuk. & V.T. Antony, Nordic J. Bot. 24(5): 541, Fig. 1. 2007.

Type: India, Kerala, Thrissur District: NH-47, Thrissur-Palakkad road at Erumbupalam, outskirts of Peechi-Vazhani wildlife sanctuary, December 23, 2003, *Joseph John Kattukunnel 4822* (CAL labeled as holotype, photo available from SSR).

Distribution in India: Kerala. **Endemic.**

Image: The species is illustrated in the original publication.

GenBank: No published sequences available.

Comments: Based on morphology, this appears to be a hybrid (H. Schafer, pers. comm. 2009). Kattuk. is the standard form of the author Joseph John Kattukunnel, who has revised Indian *Momordica* (Joseph and Antony 2010). The holotype bears the collection number 4833, not 133 as given in the protologue.

**55. *Momordica subangulata*** Blume, Bijdr. Fl. Ned. Ind. 15: 928. 1826.

Type: Java, Mt. Salak, *Blume 769* (L).

*Momordica subangulata* subsp. *renigera* (Wall. ex G. Don) W.J.de Wilde, Bot. Zhurn. (Moscow & Leningrad) 87(3): 147. 2002.

*Momordica renigera* Wall. ex G. Don, Gen. Hist. 3: 36. 1834.

Type: Myanmar, Pome hills, *Wallich Cat*. 6743 (K?).

Distribution in India: Karnataka, Kerala, Maharashtra, Meghalaya, Sikkim, Arunachal Pradesh, Assam, Manipur, Meghalaya, Mezoram, Nagaland, Sikkim, Tripura, West Bengal.

Distribution outside India: China, Bangladesh, Indonesia (Java, Sumatra), Laos, Peninsular Malaysia, Myanmar, Thailand, Vietnam.

Image: Nothing reliable found online.

GenBank: Sequences from Schaefer and Renner (2010), e.g., GQ163451, GQ163332.

Comments: Molecular data are needed to confirm that the name *Momordica renigera* described from Myanmar really applies to material from Java and India.

**56. *Neoalsomitra clavigera*** (Wall.) Hutch., Ann. Bot. (Oxford), ser. 2,6: 101. 1942.

*Zanonia clavigera* Wall., Pl. Asiat. Rar. 2: 28. t. 133. 1831.

*Alsomitra clavigera* (Wall.) M.Roem., Fam. Nat. Syn. Monogr. 2: 118. 1846, nom. nud.

Type: Bangladesh, Sylhet, *Wallich Cat. 3725A* (K).

*Neoalsomitra clavigera* (Wall.) Hutch.var. *hookeri* (C.B. Clarke) Chakrav., Rec. Bot. Surv. India 17(1): 197. 1959.

Type: Bangladesh, Sylhet, *Freire De Silva 203* (K-W, BM).

*Gynostemma integrifoliolum* Cogn. in A. & C. DC., Monogr. Phan. 3: 916. 1881. [as *integrifoliola*]

*Alsomitra integrifoliola* (Cogn.) Hayata, J. College Science, Imperial Univ. Tokyo 30(1): 121. 1911.

*Neoalsomitra integrifoliola* (Cogn.) Hutch., Ann. Bot. 6: 99. 1942

Syntypes: The Philippines, Luzon, *Cuming 767* (G-DC), Calanony, *Cuming 517* (G-BOISS).

*Alsomitra pubigera* Prain, J. As. Soc. Bengal, Pt. 2, Nat. Hist. 67: 292. 1898

Type: Myanmar, Mt. Kachin, King’s collector (herbarium?).

Distribution in India: Arunachal Pradesh, Assam, Haryana, Himachal Pradesh, Jammu & Kashmir, Manipur, Meghalaya, Sikkim, Punjab, Uttar Pradesh, West Bengal.

Distribution outside India: Bangladesh, Bhutan, Myanmar, S China (especially Yunnan and Hainan), Vietnam, Laos, Cambodia, N Sumatra, the Philippines, east to NE Australia (Queensland) and the Pacific (Solomon Island and east to Fiji); absent from the tropical everwet rain forests of Java and Borneo.

Image: Many photos of this large-fruited and large-seeded species can be found online.

GenBank: Sequences from Kocyan et al. (2007), e.g., DQ536573, DQ535830.

Comments: *Neoalsomitra* has 11 further species in Malesia, S China, New Guinea, Australia, and Fiji (De Wilde and Duyfjes 2003; Schaefer and Renner 2011a). Its phylogenetic position can be seen in Fig. 1.

**57.** ***Schizopepon bicirrhosus*** (C.B. Clarke) C. Jeffrey, Kew Bull. 34(4): 802. 1980.

*Melothria bicirrhosa* C.B. Clarke, Fl. Brit. India 2: 627. 1879.

Type: Myanmar (Burma), *Griffith 2522* (K).

*Schizopepon wardii* Chakrav., J. Bombay Nat. Hist. Soc. 50(4): 900, pl. 6. 1952.

Type: Assam, Delei Valley, alt. 11000 ft, Rhododendron-Conifer Forest, open Gullies facing north; August 23, 1928, *F. Kingdon Ward 8667* (K).

Distribution in India: Northeast India (Meghalaya, Manipur).

Distribution outside India: China (S. Xizang), Myanmar.

Image: Nothing reliable found online.

GenBank: No published sequences available.

Comments: The synonymization of *Schizopepon wardii* here follows Jeffrey (1980) and Lu et al. (2011). Chakravarty (1982) instead accepted *Schizopepon wardii* and wrote that it had “affinity towards *Schizopepon macranthus* Handel-Mazzetti, but differs in the following characters: (i) leaves not lobed (ii) pedicels longer and (iii) connective produced beyond the loculi.” Besides its four species listed here, *Schizopepon* has another five species in Russia, China, and Japan (Schaefer and Renner 2011a; Lu et al. 2011).

**58. *Schizopepon longipes*** Gagnep., Bull. Mus. Natl. Hist. Nat. 24(5): 378. 1918.

Type: China, Sechuan, near Ta-tsien-lou, *Mussot* s.n. (P).

Distribution in India: Northeast India.

Distribution outside India: China (S. Xizang), Myanmar.

Image: Nothing reliable found online.

GenBank: No published sequences available.

Comment: The *Flora of Bhutan* (2(1): 260. 1991) records this species from Bhutan and Darjeeling in West Bengal. Jeffrey (1980, 1982) changed his mind about Indian material that he first identified as *Schizopepon dioicus* Cogn., but later as *Schizopepon longipes*.

**59. *Schizopepon macranthus*** Handel-Mazzetti, Symb. Sin. 7(4): 1064. 1936.

Type: China, Sichuan, Muli, Lijiacun, 2850-3000 m, 23 July 1915

*Handel-Mazzetti 7153* (B, destroyed?).

Distribution in India: Possibly Northeast India.

Distribution outside India: China (W Sichuan and NW Yunnan).

Image: Nothing reliable found online.

GenBank: No published sequences available.

Comment: Jeffrey (1980) does not mention this species, while Chakravarty (1982) discusses its similarity to *Schizopepon wardii*, here considered a synonym of *Schizopepon bicirrhosus*. The *Flora of China* (Lu et al. 2011), recognizes it as a distinct species.

**60. *Sicyos edulis*** Jacq., Enum. Syst. Pl. 32. 1760.

*Sechium edule* (Jacq.) Sw., Fl. Ind. Occid. 2(2): 1150. 1800.

Type: “In insulis Caribaeis vicinaque Americes continente detexit novas.”

*Sechium americanum* Poir., Encycl. (Lamarck) 7: 50. 1806.

Type: “Cette planté croît naturellement à la Jamaique, où on la cultive aussi à cause de ses fruits que l’on mange, & qui s’imploient dans les ragouts.”

Distribution in India: Cultivated throughout India.

Distribution outside India: Native to Mexico, cultivated throughout the tropics.

Image: http://www.flowersofindia.net/catalog/slides/Chaco.html

GenBank: Sebastian et al. (2012) and numerous other sequences.

Comments: Molecular data show that *Sechium* is embedded within the genus *Sicyos* (Sebastian et al. 2012).

**61. *Siraitia sikkimensis*** (Chakrav.) C. Jeffrey, Kew Bull. 36(4): 737. 1982.

*Neoluffa sikkimensis* Chakrav., J. Bombay Nat. Hist. Soc. 50(4): 895, pl. 3. 1952.

Type: India, Sikkim Himalaya, near Sittong, alt. 1500 ft, 12 May 1876, *G. King s.n*. (CAL, 3 sheets, photos available from SSR)

Distribution in India: Sikkim, West Bengal.

Distribution outside India: China (S Yunnan).

Image: Nothing reliable found online.

GenBank: No published sequences available.

Comments: The genus *Siraitia* has five species, four in India, Indonesia, Peninsular Malaysia, Thailand, South and Southwest China, and one Southern Tanzania and Southeast Nigeria (Schaefer and Renner, 2011a, b). The cucurbitane-type triterpene glycoside constituents of *Siraitia grosvenorii* are the source of plant-derived sweeteners.

**62. *Solena amplexicaulis*** (Lam.) Gandhi in Saldanha & Nicolson, Fl. Hassan Distr. 179. 1976.

*Bryonia amplexicaulis* Lam., Encycl. 1: 496. 1785.

*Karivia amplexicaulis* (Lam.) Arn., J. Bot. 3: 275. 1841.

*Melothria amplexicaulis* (Lam.) Cogn. in A. & C. DC., Monogr. Phan. 3: 621. 1881.

Type: S India, *Sonnerat* s.n. (P-LAM).

Distribution in India: Tamil Nadu, Karnataka, Kerala. **Endemic.**

Images: efloraofindia at https://sites.google.com/site/efloraofindia/species/a---l/cl/cucurbitaceae/solena/solena-amplexicaulis

GenBank: Sequences from Chen et al. (2010), e.g., GQ436395, GQ435029.

Comments: Following De Wilde and Duyfjes (2004c), *Solena* comprises three or four species while in the past, only one species, *Solena amplexicaulis*, was recognized, which supposedly ranged from NE Afghanistan through India and Sri Lanka. Based on several vegetative and reproductive differences, De Wilde and Duyfjes instead recognize *Solena amplexicaulus* from South India, *Solena umbellata* from South India and Sri Lanka, and *Solena heterophylla* with two subspecies, one from NE Afghanistan eastward, the other in N India and east to China. The *Flora of China* (Lu et al. 2011) follows this treatment.

**63. *Solena heterophylla*** Lour., Fl. Cochinch. 2: 514. 1790.

subsp. **heterophylla**

*Melothria heterophylla* (Lour.) Cogn. in A. & C. DC., Monogr. Phan. 3: 618. 1881.

Type: Vietnam, *Loureiro* s.n. (BM http://plants.jstor.org/specimen/bm000944657 ). *Bryonia rheedei* Blume, Bijdr. Fl. Ned. Ind. 15: 925. 1826

*Karivia rheedei* (Blume) M.Roem., Fam. Nat. Syn. Monogr. 2: 45. 1846

Type: Java, *Blume* s.n. (L, Barcode: L0127474).

*Bryonia sagittata* Blume, Bijdr. Fl. Ned. Ind. 15: 925. 1826.

Type: Java, *Blume* s.n. (L, Barcode: L0127475).

*Melothria ovata* Cogn. in Engl. Pflanzenr. IV. 275, 1 (Heft 66): 114. 1916.

Type: India, Sikkim, near Labdah, 650 m a.s.l., Aug. 1884, *collector unknown* (G-BOISS).

Distribution in India: Widely distributed all over India (Chakravarty 1982).

Distribution outside India: NE Afghanistan, Indonesia (Java), Peninsular Malaysia, Myanmar, Nepal, Thailand, Vietnam (Lu et al. 2011).

GenBank: Sequences from Kocyan et al. (2007), e.g., DQ536737, DQ536870.

Comments: See under *Solena amplexicaulis*.

subsp. **napaulensis** (Ser.) W.J.de Wilde & Duyfjes, Blumea 49(1): 75. 2004.

*Bryonia napaulensis* Ser., Prodr. 3: 307. 1828.

*Zehneria umbellata* (Klein ex Willd.) Thwaites var. *napaulensis* (Ser.) C.B. Clarke, Fl. Brit. India 2: 625. 1879.

Type: Nepal, *Wallich* s.n. (G).

Distribution in India: Western Himalaya (Garhwal, Kumaon hills, Uttarakhand).

Distribution outside India: China (Yunnan), Myanmar, Nepal (Lu et al. 2011).

GenBank: No published sequences available.

Comments: See under *Solena amplexicaulis*.

**64. *Solena umbellata*** (Willd.) W.J. de Wilde & Duyfjes, Blumea 49(1): 77. 2004.

*Bryonia umbellata* Willd., Sp. Pl., ed. 4(1): 618. 1805.

*Momordica umbellata* (Willd.) Roxb., Hort. Bengal. 79. 1832.

*Karivia umbellata* (Willd.) Arn., J. Bot. 3: 275. 1841.

*Zehneria umbellata* (Willd.) Thwaites, Enum. Pl. Zeyl. 2: 125. 1859.

Type: South India, *J. G. Klein 765* (lecto B-W), designated by De Wilde and Duyfjes (2004).

*Melothria angulata* Chakrav., J. Bombay Nat. Hist. Soc. 50(4): 899. 1952.

*Zehneria angulata* (Chakrav.) J. L. Ellis, Bull. Bot. Surv. India 9(1-4): 8. 1968 (“1967”).

*Solena angulata* (Chakrav.) Babu, Herb. Fl. Dehra Dun 203. 1977.

Type: South India, Gomata, alt. 5500 ft, *Malcolmpeth 81* (CAL photo available from SSR).

Distribution in India: Goa, Karnataka, Kerala, Tamil Nadu.

Distribution outside India: Sri Lanka.

Image: Nothing reliable found online.

GenBank: No published sequences available.

Comments: The genus *Melothria* is restricted to tropical Central and South America, where it has about 12 species (Schaefer and Renner, 2011a). Based on molecular data, the Asian species formerly assigned to *Melothria* belong in *Cucumis*, *Solena*, and other genera. For the number of species of *Solena* see comment under *Solena amplexicaulis*.

**65. *Thladiantha hookeri*** C.B. Clarke, Fl. Brit. India 2(6): 631. 1879.

Syntypes: India, Meghalaya [Assam], *Griffith* s.n. (K). Khasia Hills, alt. 4000–6000 ft; *J.D. Hooker & Thomson*s.n. (CAL photo available from SSR, K). Myrung and Nunklow, *J.D. Hooker & Thomson*s.n. (K).

*Thladiantha hookeri* C.B. Clarke var. *palmatifolia* Chakrav., Notes Roy. Bot. Gard. Edinburgh 20(48): 122. 1948.

Type: India, Manipur [Assam], Kala Naga Hills, *Watt 7306* (E).

*Hemsleya trifoliolata* Cogn., Repert. Spec. Nov. Regni Veg. 6(15/20): 304. 1909. *Thladiantha hookeri* forma *trifoliolata* (Cogn.) Chakrav., Notes Roy. Bot. Gard. Edinburgh 20: 122. 1948 = *Thladiantha hookeri* var. *irregularis* Chakrav., Fasc. Fl. India 11: 104. 1982, nom. nov.

Type: China, Yunnan, A. *Henry 12295D* (Z).

*Thladiantha pentadactyla* Cogn. in Engl. Pflanzenr. IV. 275, 1 (Heft 66): 52. 1916.

Type: China, Yunnan, alt. 1700 m, *A. Henry 12295D* (B), same type as previous name.

*Thladiantha heptadactyla* Cogn. in Engl. Pflanzenr. IV. 275, 1 (Heft 66): 52. 1916.

Type: China, Yunnan, Lou Kong, alt. 2800m, May 1886, *Delavay* s.n. (P).

Distribution in India: Assam, Manipur, Meghalaya, Nagaland.

Distribution outside India: China (Yunnan), Bhutan, Laos, Myanmar, Thailand, Vietnam.

Image: http://apps.kew.org/herbcat/getImage.do?imageBarcode=K000036903

GenBank: Sequences from Kocyan et al. (2007) and Li et al. (2011), e.g., JF978932, DQ536601.

Comments: *Thladiantha* has c. 30 species in China, Taiwan, Tibet, India, Korea, Japan, Thailand, Vietnam, Indonesia, Philippines, and New Guinea.

**66.** ***Thladiantha cordifolia*** (Blume) Cogn. in A. & C. DC., Monogr. Phan. 3: 424. 1881. *Luffa cordifolia* Blume, Bijdr. Fl. Ned. Ind. 15: 929. 1826.

Type: Java, *Blume 1464*, fruit (lectotype L, barcode L0001624, designated by De Wilde and Duyfjes (2006); isotype L; CAL has two sheets without collection numbers).

*Thladiantha calcarata* (Wall.) C.B. Clarke, J. Linn. Soc., Bot. 15: 126. 1876, nom. nud.

*Momordica calcarata* Colebr. ex Wall., Cat. No. 6740. 1832, nom. nud.

*Thladiantha calcarata* (Wall.) C.B. Clarke [nom. nud.] var. *subglabra* Cogn. in A. & C. DC., Monogr. Phan. 3: 424. 1881. (Listed as “*Thladiantha cordifolia* (Blume) Cogn. var. *subglabra* Cogn.” by Chakravarty, 1982.)

Type: India, Meghalaya, Khasia, 1300 m, *J.D. Hooker & T. Thomson 1* (CAL 2 sheets, photos available from SSR, K).

Distribution in India: Andhra Pradesh, Arunachal Pradesh, Assam, Manipur, Meghalaya, Nagaland, Sikkim, Tamil Nadu, Tripura, West Bengal.

Distribution outside India: Nepal, China (Guangdong, Guangxi, Sichuan, Yunnan), Indonesia (Java, Sumatra), Laos, Peninsular Malaysia, Myanmar, Thailand, Vietnam.

Image: efloraofindia at https://sites.google.com/site/efloraofindia/species/a---l/cl/cucurbitaceae/thladiantha/thladiantha-cordifolia

GenBank: Sequences from Schaefer and Renner (2010) and Li et al. (2011), e.g., JF978906, GQ163340.

Comments: Further synonmys are given in Lu et al. (2011).

**67. *Trichosanthes anaimalaiensis*** Bedd., Madras J. Lit. Sci. 3,1: 47. 1864.

Type: India, Tamil Nadu, Anaimalai Mts., *Beddome 3234* (BM http://plants.jstor.org/specimen/bm000885793 )

*Trichosanthes bracteata* (Lam.) Voigt var. *tomentosa* (C.B. Clarke) Chakrav., Rec. Bot. Surv. India 17(1): 47. 1959, nom. illeg., because its type, *Abdul Khalil*s.n. (CAL photo available from SSR) from Myanmar, Southern Shan State, Indine, is a syntype of *Trichosanthes burmensis* Kundu (see under *Trichosanthes rubriflos*).

*Trichosanthes palmata* L. var. *tomentosa* Heyne ex C.B. Clarke, Fl. Brit. India 2(6): 607. 1879.

Syntypes: India, Deccan Peninsular Mountains; *Wight no. 1134 (* HBG online at JSTOR*), 1136 partly*, *G. Thomson*s.n.; Sri Lanka, alt. 2600 ft, *Gardner* s.n. (K).

Distribution in India: Andaman & Nicobar Islands (Naithani 1990), Andhra Pradesh, Arunachal Pradesh, Karnataka, Kerala, Maharashtra, Tamil Nadu, Tripura. **Endemic.**

Image: Nothing reliable found online.

GenBank: No published sequences available.

**68. *Trichosanthes bracteata*** (Lam.) Voigt, Hort. Suburb. Calcutt. 58. 1845.

*Modecca bracteata* Lam., Encycl. 4: 210. 1797.

Type: India, *Sonnerat* s.n. (P-LAM).

Distribution in India: Peninsular India, Khasia Hills, Dehra Doon, Bengal.

Distribution outside India: China (Guizhou), Nepal (? see comments).

Image: Nothing reliable found online.

GenBank: Sequences from De Boer et al. (2012) from Indian material, e.g., HE661317, HE661484.

Comments: Jeffrey (1980) and Lu et al. (2011) treat *Trichosanthes bracteata* as a synonym of *Trichosanthes tricuspidata*, which ranges from China (Guizhou), Peninsular Malaysia, Nepal, Thailand, to Vietnam, while Chakravarty (1982) recognized *Trichosanthes bracteata* with two varieties, var. *bracteata* from throughout India, Myanmar, China, and Australia, and var. *tomentosa* (an illegitimate name here treated under *Trichosanthes anaimalaiensis*) on the Andaman and Nicobar islands, and in Arunachal Pradesh, Karnataka, Kerala, Maharashtra, Tamil Nadu, Tripura, as well as Myanmar and Java. Another species concept is that of De Wilde and Duyfjes (2008a, 2010).

**69. *Trichosanthes cordata*** Roxb. Fl. Ind. 3: 703 1832.

Type: Bangladesh, mouth of the river Meghna, *Wallich Cat. No*. 6686A (K, CAL).

*Trichosanthes macrosiphon* Kurz, J. Asiat. Soc. Bengal, Pt. 2, Nat. Hist. 41: 308. 1872.

Type: Myanmar, Tenasserim, *W.S. Kurz* (CAL, no image seen).

Distribution in India: Andhra Pradesh, Arunachal Pradesh, Assam, Bihar, Chhattisgarh, Jharkhand, Madhya Pradesh, Manipur, Meghalaya, Mizoram, Nagaland, Rajasthan, Sikkim, Tamil Nadu, Tripura, Uttar Pradesh, Uttarakhand, West Bengal

Distribution outside India: Bangladesh, Bhutan, China, Myanmar, Nepal.

Image: Nothing reliable found online.

GenBank: No published sequences available.

Comments: C.B. Clarke (1879: 608) synomized *Trichosanthes macrosiphon* under *Trichosanthes cordata* Roxb. because the protologue does not contain anything uniquely distinctive compared to the protologue of *Trichosanthes cordata*. The personal herbarium of Wilhelm Sulpiz Kurz is at CAL, but that we have not received the requested type image.

**70. *Trichosanthes costata*** Bl., Bijdr. Fl. Ned. Ind. 15: 933. 1826.

Type: Java, *Blume* s.n. (L, barcode L0589632), designated by De Wilde and Duyfjes (2006).

*Gymnopetalum chinense* (Lour.) Merr., Philipp. J. Sci. 15: 256. 1919.

*Euonymus chinensis* Lour., Fl. Cochinch. 1: 156. 1790 (as *Evonymus*).

Type: Untraced. Neotype: South China, *Levine 1705* (holotype A, designated by De Wilde and Duyfjes 2008b).

*Tripodanthera cochinchinensis* (Lour.) M.Roem., Fam. Nat. Syn. Monogr. 2: 48. 1846.

*Gymnopetalum cochinchinense* (Lour.) Kurz, J. Asiat. Soc. Bengal, Pt. 2, Nat. Hist. 40: 57. 1871.

*Bryonia cochinchinensis* Lour., Fl. Cochinch. 2: 595. 1790.

Type: Vietnam, *Loureiro 595* (BM, http://plants.jstor.org/specimen/viewer/bm000944642 ).

*Momordica tubiflora* Roxb., Fl. Ind. 3: 711. 1832.

*Scotanthus tubiflorus* (Roxb.) Naudin, Ann. Sci. Nat., Bot. sér. 4, 16: 172, f. 3. 1862, nom. superfl.

Type: India, *Wallich Cat. 6749* (K).

*Gymnopetalum quinquelobum* Miq., Fl. Ned. Ind. 1: 681. 1855.

Type: Java, Soerakarta, *T. Horsfield*s.n. (BM image seen)

*Gymnopetalum heterophyllum* Kurz, J. Bot. 13: 326. 1875.

Type: Kamorta Island (part of the Nicobar Islands); *Wallich Cat. 6711* (K).

Distribution in India: Andaman & Nicobar Islands, Andhra Pradesh, Arunachal Pradesh, Assam, Bihar, Manipur, Meghalaya, Sikkim, Tripura, Uttar Pradesh, West Bengal.

Distribution outside India: China, Java, Myanmar, Sri Lanka, Vietnam.

Images: Photos available upon request from A. Pandey or SSR.

GenBank: Schaefer et al. (2008), most sequences under the name *Gymnopetalum chinense*, e.g., HE661294, HQ201978.

Comments: Based on molecular data, *Gymnopetalum chinense* belongs in the genus *Trichosanthes* (De Boer et al. 2012; contra De Wilde and Duyfjes 2006c). In *Trichosanthes*, however, the epithet *chinense* is already occupied by *Trichosanthes chinensis* Ser. (1828). The second name in line of priority would be *Gymnopetalum cochinchinensis*, based on the basionym *Bryonia cochinchinensis*. However, the combination *Trichosanthes cochinchinensis* (Lour.) M.Roem. (based on *Trichosanthes cucumerina* Lour.) blocks that transfer, too. The third available name is *Trichosanthes costata* Blume, and this name must be used for *Gymnopetalum chinense* if the species is placed in *Trichosanthes* (De Boer and Thulin 2012).

**71. *Trichosanthes cucumerina*** L., Sp. Pl. 2: 1008. 1753.

Lectotype: India, Kerala, “Padavalam” in Rheede, Hort. Malab. 8: 29. t. 15. 1688, designated by Keraudren-Aymonin in Aubréville & Leroy (ed.), Fl. Cambodge Laos Viêt-Nam 15: 91. 1975.

*Trichosanthes anguina* L., Sp. Pl. 2: 1008. 1753.

Lectotype: China, “Anguina Sinensis, flore albo, elegantissimo, capillamentis tenuissimis ornato, fructu longo intorto, sub initium ex albo, & viridi variegato, per maturitatem prorsus rubro” in Micheli, Nov. Pl. Gen. 12. t. 9. 1729, designated by Jeffrey in Jarvis & al. (ed.), Regnum Veg. 127: 95. 1993.

*Cucumis anguinus* L., Sp. Pl., ed. 10. 2: 1279. 1759.

Type: “Habitat [in India.], Sp. Pl., ed. 2, 2: 1438. 1763.” Lectotype: “Petola Anguina” in Rumphius, Herb. Amboin. 5: 407. t. 148, 1747, designated by Merrill in Interpret. Rumph. Herb. Amb. 494. 1917.

*Trichosanthes pachyrrhachis* Kundu, J. Bot. 77: 9. 1939.

Syntypes: Northwest India, 1844, *M.P. Edgeworth 63* (K), Mangalor, 1847, *R.F. Hohenacker* (herbarium?), synonymized here by Chakravarty (1959) and Jeffrey (1980).

*Trichosanthes brevibracteata* Kundu, J. Bot. 77: 10. 1939.

Paratypes (Art. 9.4): India, Karnal, Punjab, 1885-1888, *J.R. Drummond 25031* (herbarium?), Ahmedabad, July 1920, *L.J. Sedgwick* (herbarium?), NW India, *Thomson* s.n. (herbarium?); synonymized here by Chakravarty (1959) and Jeffrey (1980).

*Trichosanthes brevibracteata* Kundu var. *sublobata* Kundu, J. Bot. 77: 11. 1939.

Type: India, Nagpur-Wardha, C.P., Sep. 2012, *Haines* (K).

*Trichosanthes brevibracteata* Kundu var. *longirostrata* Kundu, J. Bot. 77: 11. 1939.

Type: Myanmar, 15 Aug. 1908, *J. H. Lace 6335* (K).

Distribution in India: Native and cultivated throughout India.

Distribution outside India: Sri Lanka and tropical China through Malesia into W, N, and NE Australia.

Image: http://www.flowersofindia.net/catalog/slides/Snake%20Gourd.html

http://apps.kew.org/herbcat/getImage.do?imageBarcode=K000742697

http://apps.kew.org/herbcat/getImage.do?imageBarcode=K000742699

GenBank: Sequences from Schaefer and Renner (2011b) and De Boer et al. (2012), e.g., HE661410, HE661486.

Comments: Widely cultivated for its edible fruits (Duyfjes and Pruesapan 2004). In 1959, Chakravarty synonymized *Trichosanthes pachyrrhachis* Kundu and

*Trichosanthes brevibracteata* Kundu under *Trichosanthes cucumerina*, but his 1982 checklist omitted both names.

**72. *Trichosanthes cucumeroides*** (Ser.) Maxim.,Franch. & Sav. Enum. Pl. Jap. 1: 172. 1873.

*Bryonia cucumeroides* Ser., Prodr. 3: 308. 1828.

*Trichosanthes ovigera* subsp. *cucumeroides* (Ser.) C. Jeffrey, Mansfeld’s Encycl. 3: 1528. (6: 2825). 2001.

Type: “Patria ignotus, Seringe manuscript” perhaps a Wallich specimen (herbarium?).

*Trichosanthes dicaelosperma* C.B. Clarke, Fl. Brit. India 2: 609. 1879.

*Trichosanthes cucumeroides* var. *dicaelosperma* (C.B. Clarke) S. K. Chen, Bull. Bot. Res., Harbin 5(2): 118. 1985.

Syntypes: India, Sikkim, J.D. Hooker s.n. (K), Khasia Mts., *Hooker & Thomson* (CAL photos available from SSR, K).

Distribution inside India: Meghalaya, Sikkim, Uttar Pradesh, West Bengal.

Distribution outside India: Guangxi, SE Xizang.

Image: Many images of this much-cultivated species can be found online.

GenBank: Several sequences, e.g., HQ829602, HQ829602.

Comment: Jeffrey (in Lu et al. 2011) prefers to treat *Trichosanthes cucumeroides* as a synonym of *Trichosanthes pilosa* Lour. (Fl. Cochinch. 2: 588. 1790). In his 1980 checklist, he does not list *Trichosanthes cucumeroides* and treats *Trichosanthes dicaelosperma* as a synonym of *Trichosanthes ovigera*. Lu et al. (2011) instead recognize *Trichosanthes cucumeroides*, with *Trichosanthes dicaelosperma* as one of its varieties.

**73. *Trichosanthes dioica*** Roxb., Fl. Ind. 3: 701. 1832.

Type: India, West Bengal, “It is much cultivated by the natives about Calcutta, during the rains.” Ic. Roxb. Lectotype?

*Trichosanthes dioica* Roxb. var. *sagittifolia* Chakrav., Rec. Bot. Surv. India 17(1): 55. 1959.

Type: Northwest India, without precise locality, *cult. (Stewart 1228)* (E).

Distribution in India: Arunachal Pradesh, Assam, Bihar, Delhi, Himachal Pradesh, Jammu & Kashmir, Meghalaya, Punjab, Rajasthan, Uttar Pradesh, West Bengal Distribution outside India: Bangladesh, Myanmar, Nepal, Pakistan, Sri Lanka.

Image: efloraofindia at https://sites.google.com/site/efloraofindia/species/a---l/cl/cucurbitaceae/trichosanthes/trichosanthes-dioica

GenBank: Sequences from Ali, Pandey, and Lee (2009) and De Boer et al. (2012), e.g., GQ240881, HE661322.

Comments: The female gametophytes were studied by Pandey et al. (1997, 2003) and pollen germination behavior by Kumari et al. (2009). The synonyimization of var. *sagittifolia* follows Jeffrey (1980).

**74. *Trichosanthes dunniana*** H. Lév., Repert. Spec. Nov. Regni Veg.10: 148. 1911.

Type: China, Guizhou, *Esquirol* 726, (E, K).

*Trichosanthes majuscula* (C.B. Clarke) Kundu, J. Bot. 77: 12. 1939.

*Trichosanthes multiloba* Miq. var. *majuscula* C.B. Clarke, Fl. Brit. India 2(6): 608. 1879.

*Trichosanthes wallichiana* (Ser.) Wight var. *majuscula* (C.B. Clarke) Cogn. in A. & C. DC., Monog. Phan. 3: 369. 1881.

Type: India, Meghalaya, Khasia Hills, alt. 4000 ft., *J.D. Hooker & Thomson s.n. (Herb. Ind. Or. Trichosanthes sp. 7)* (K).

*Trichosanthes prazeri* Kundu, J. Bombay Nat. Hist. Soc. 43(2): 378. 1942.

Type: Upper Myanmar, May 1888, Khoni, *J.C. Prazer*s.n. (CAL, 3 sheets, photos available from SSR).

Distribution in India: Minimally Meghalaya.

Distribution outside India: China, Myanmar, Thailand.

Image: Some of the type specimens can be found online.

GenBank: Several sequences.e.g., HQ829503, HQ829605.

Comments: The acceptance of *Trichosanthes dunniana* for India and the synonymization of *Trichosanthes majuscula* follow Jeffrey (1982). Chakravarty (1959) recognized *Trichosanthes majuscula*, saying that the species required further examination. The leaves are larger than in *Trichosanthes wallichiana* proper; otherwise it closely agrees with that species.

**75. *Trichosanthes kerrii*** Craib, Bull. Misc. Inform. Kew. 1914: 7. 1914.

Type: North Thailand, *Kerr* 2454 (BM, K).

*Trichosanthes tomentosa* Chakrav., J. Bombay Nat. Hist. Soc. 50(4): 894, f. 45. 1952.

Type: India, Nagaland, Kohima and Naga Hill, alt. 4500 ft; 22 May 1895; *Watt 11640* (CAL, 3 sheets, photos available from SSR).

Distribution in India: Nagaland, Mongsemdi Naga hills (Chakravarty, 1982).

Distribution outside India: China (SW Yunnan), Laos, N Thailand, N Vietnam.

Image: See type images.

GenBank: Sequences from Schaefer et al. (2008) and De Boer et al. (2012), e.g., HE661333, HE661498.

Comments: Jeffrey (1982), Duyfjes and Pruesapan (2004), and Lu et al. (2011) all list *Trichosanthes tomentosa* as a synonym of *Trichosanthes kerrii*.

**76. *Trichosanthes khasiana*** Kundu, J. Bot. 77: 11. 1939.

Type: India, Meghalaya, Khasia Hills, Hooker & Thomson (K, http://apps.kew.org/herbcat/getImage.do?imageBarcode=K000102020 )

Distribution in India: Meghalaya.

Distribution outside India: **Endemic.**

Image: Nothing found online other than the type image.

GenBank: No published sequences available.

Comments: Jeffrey (1982) and De Boer and Thulin (2012) recognize this species as distinct.

**77. *Trichosanthes lepiniana*** (Naudin) Cogn. in A. & C. DC., Monogr. Phan. 3: 377. 1881.

*Involucraria lepiniana* Naudin in Huber, Cat. 11. 1868.

Syntypes?: India, Union Territory, Pondichery, *Trichosanthes Lepine* s.n. (P http://plants.jstor.org/specimen/bm000900967 ); Sikkim, *J.D. Hooker & Thomson 14* (K, P).

Distribution in India: Union Territory.

Distribution outside India: Unclear, see comments.

Image: Nothing found online other than the type image.

GenBank: Sequences from De Boer et al. (2012) from Nepalese and Chinese material, e.g., HE661507, HE661341.

Comments: Jeffrey (1980) initially considered *Trichosanthes lepiniana* a synonym of *Trichosanthes tricuspidata*, but he later (1982) recognized it as a separate species, as did Chakravarty (1982).

**78. *Trichosanthes lobata*** Roxb., Fl. Ind. 3: 703. 1832.

Type: India, “This plant grows in hedges, and among bushes.” *Roxburgh 992* (K)

*Trichosanthes perrottetiana* Cogn. in A. & C. DC., Monogr. Phan. 3: 362. 1881.

Type: India, Union Territory, Pondichery, *Perrottet 256* (G-BOISS, W).

Distribution in India: Andhra Pradesh, Karnataka, Kerala, Puducherry, Tamil Nadu, Uttar Pradesh, West Bengal.

Distribution outside India: China.

Image: Nothing found online.

GenBank: No published sequences available.

Comments: Chakravarty (1982) recognizes both *Trichosanthes lobata* and *Trichosanthes perrottetiana*, whileJeffrey (1980) synonoymizes T. *perrottetiana* (and also *Trichosanthes villosula*) under *Trichosanthes lobata*. We have followed Lu et al. (2011) in maintaining *Trichosanthes villosula* separate.

**79. *Trichosanthes nervifolia*** L., Sp. Pl. 2: 1008. 1753.

Lectotype: India, Kerala, Tota-piri, in Rheede, Hort. Malab. 8: 33, t. 17. 1688, designated by Majumdar & Bakshi in Taxon 28: 354. 1979.

*Trichosanthes cuspidata* Lam., Encycl. 1: 190. 1783.

Type: India, Rheede, Hort. Malab. 8; 31, t. 16.

Distribution in India: Andhra Pradesh, Arunachal Pradesh, Assam, Bihar, Goa, Karnataka, Kerala, Madhya Pradesh, Manipur, Meghalaya, Mizoram, Nagaland, Rajasthan, Sikkim, Tamil Nadu, Tripura, Uttar Pradesh, West Bengal.

Distribution outside India: Sri Lanka.

Image: Nothing found online.

GenBank: Sequences from De Boer et al. (2012) from Sri Lankan material, e.g., HE661514, HE661350.

**80. *Trichosanthes ovigera*** Blume, Bijdr. Fl. Ned. Ind. 15: 934. 1826.

Type: Java, Gunung Salak, *Blume* s.n. (L barcodes L0130442, L0130439 , P).

*Trichosanthes ovigera* Blume var. *sikkimensis* Kundu, J. Bombay. Nat. Hist. Soc. 43(3): 383. 1942.

Type: India, Selim, Sikkim, 1000 feet, Oct. 1884, *C.B. Clarke* (CAL). Other cited sheets: India, Rungtung, Sikkim Dec. 1876, *A.B*. (King’s Collector) (CAL); Sikkim, 5000 feet, 23 Sep. 1875, *G. King* (CAL?); Runjeet, Darjeeling, Sep. 1884, *C.B. Clarke* (CAL); Kobo, Abor Expedition, Assam. Dec. 1911, *J.H. Burkill 37420* (K).

*Trichosanthes horsfieldii* Miq., Fl. Ned. Ind. 1: 677. 1855.

Type: Java, Priangan, *T. Horsfield 15* (BM, K, U).

*Trichosanthes himalensis* C.B. Clarke, Fl. Brit. India 2(6): 608. 1879.

Type: India, Sikkim, alt. 2000-5000 ft, from Yoksun to the plains; *J.D. Hooker, C.B. Clarke*s.n. (K).

*Trichosanthes himalensis* var. *glabrior* C.B. Clarke, Fl. Brit. India 2(6): 608. 1879.

Type: India, Meghalaya, Khasia, alt. 4000 ft, *Trichosanthes sp. 9 in J.D. Hooker & T. Thomson*s.n. (K).

*Trichosanthes himalensis* var. *indivisa* Chakrav., Rec. Bot. Surv. India 17(1): 51. 1959, nom. illeg. Sikkim, 3500 feet, 11 Dec. 1877, *G. King* (CAL, photo available from SSR)

*Trichosanthes himalensis* var. *sikkimensis* (Kundu) Thoth., Bull. Bot. Surv. India 2(1&2): 169. 1960.

Distribution in India: Andaman & Nicobar Islands, Arunachal Pradesh, Assam, Meghalaya, Sikkim, Tripura, Uttar Pradesh, West Bengal.

Distribution outside India: Australia, Bangladesh, China, Japan, Java, Myanmar, Nepal.

Image: A few of the type specimens can be found online.

GenBank: Sequences from Kocyan et al. (2007) and Schaefer et al. (2008) from Japanese and Australian material, e.g., DQ536604, DQ536875.

Comments: The list of synonyms of *Trichosanthes ovigera* follows Jeffrey (1980) except for *Trichosanthes dicaelosperma*, which he also synonymizes here, while we have followed Lu et al. (2011) who consider *Trichosanthes dicaelosperma* one of the varieties of *Trichosanthes cucumeroides*. Lu et al. (2011) and De Wilde and Duyfjes (2008a, b) both consider *Trichosanthes ovigera* a synonym of *Trichosanthes pilosa*. Morphological and molecular work is needed to clarify species boundaries in *Trichosanthes*.

**81. *Trichosanthes rubriflos*** Thorel ex Cayla, Bull. Mus. Natl. Hist. Nat. 14: 170. 1908.

*Trichosanthes pubera* Blume subsp. *rubriflos* (Thorel ex Cayla) Duyfjes & Pruesapan, Thai Forest Bull., Bot. 32: 94. 2004.

Lectotype: Cambodia, Stung-streng, *Thorel* 2126 (K, P), designated by Keraudren (1975).

*Trichosanthes burmensis* Kundu, J. Bombay Nat. Hist. Soc. 43(2): 381. 1942.

Syntypes: Upper Myanmar, Southern Shan State, Indine, 1893, *Abdul Khalil*s.n. (CAL, 3 sheets, photos available from SSR), Pegu, *W.S. Kurz 1062* (CAL, no image seen).

Distribution in India: Unknown.

Distribution outside India: China, Myanmar, Cambodia, Thailand.

GenBank: Sequences from De Boer et al. (2012) based on material from Thailand, mostly under *Trichosanthes pubera* subsp. *rubriflos*, e.g., HE661533, HE661451.

Comments: In his 1980 checklist of the Indian Cucurbitaceae, Jeffrey recognized *Trichosanthes rubriflos* with two doubful synonyms, *Trichosanthes prazeri* Kundu and *Trichosanthes burmensis* Kundu, but in 1982, he moved *Trichosanthes prazeri* into the synonymy of *Trichosanthes dunniana*. Chakravarty (1959, 1982) also listed *Trichosanthes rubriflos* for India, but kept *Trichosanthes majuscula* and *Trichosanthes prazeri* separate, and considered *Trichosanthes burmensis* a synonym of *Trichosanthes bracteata*, which is in error. Duyfjes and Pruesapan (2004) considered *Trichosanthes rubriflos* a subspecies of *Trichosanthes pubera* Blume, described from Java. According to the *Trichosanthes* expert Hugo De Boer (pers. comm. 24 Oct. 2012), the holotype of *Trichosanthes burmensis*, *Abdul Khalil*s.n., resembles material of *Trichosanthes rubriflos* from Thailand, and the label notes that the flowers are red. Another form of *Trichosanthes* described by Kundu, *Trichosanthes burmensis* var. *alba* Kundu is a synonym of *Trichosanthes tricuspidata* (see below).

**82. *Trichosanthes quinquangulata*** A. Gray, U.S. Expl. Exped., Phan. 1: 645. 1854.

Type: Philippines, Mangsee, *Wilkes s. n. 1842/2* (US).

Distribution in India: Andaman Islands (voucher: L. *Rasingam 17583*, PBL).

Distribution outside India: South China, Myanmar, Thailand, Vietnam, Cambodia, Laos, Peninsular Malaysia, Singapore, Indonesia (Sumatra, Borneo, Java, Moluccas, New Guinea (West Papua and Papua New Guinea, Philippines.

Image: Nothing reliable found online.

GenBank: Sequences from De Boer et al. (2012), e.g., HE661535, HE661375.

Comment: The occurrence of this species on the Andaman Islands is a discovery of Rasingam (2012).

**83. *Trichosanthes scabra*** Lour., Fl. Cochinch. 2: 589. 1790.

*Gymnopetalum scabrum* (Lour.) W.J.de Wilde & Duyfjes, Reinwardtia 12: 268. 2008.

Type: Vietnam, Annam, *Poilane 11322* (neotype P; isoneotype L), designated by De Wilde and Duyfjes (2008b).

*Cucumis integrifolius* Roxb., Fl. Ind. 3: 724. 1832.

*Gymnopetalum integrifolium* (Roxb.) Kurz, J. Asiat. Soc. Bengal, Pt. 2, Nat. Hist.40: 58. 1871.

*Trichosanthes integrifolia* (Roxb.) Kurz, J. Asiat. Soc. Bengal, Pt. 2, Nat. Hist. 46: 99. 1877.

Type: Myanmar (Burma), *Wallich Cat. 6730* (K-W).

*Gymnopetalum integrifolium* (Roxb.) Kurz var. *pectinatum* W.J.de Wilde & Duyfjes, Blumea 51: 287. 2006.

*Gymnopetalum scabrum* (Lour.) W.J.de Wilde & Duyfjes var. *pectinatum* (W.J. de Wilde& Duyfjes) W.J.de Wilde & Duyfjes, Reinwardtia 12: 268. 2008.

*Trichosanthes scabra* Lour. var. *pectinata* (W.J.de Wilde & Duyfjes) H.J.De Boer, Phytokeys 12: 30. 2012.

Type: Java, Indonesia, *W.J. de Wilde and Duyfjes 21692* (L).

*Gymnopetalum penicaudii* Gagnep. (1918) Bull. Mus. Natl. Hist. Nat. 24: 374.

*Gymnopetalum scabrum* (Lour.) W.J.de Wilde & Duyfjes var. *penicaudii* (Gagnep.) W.J.de Wilde& Duyfjes, Reinwardtia 12: 268. 2008.

*Trichosanthes scabra* Lour. var. *penicaudii* (Gagnep.) H.J.De Boer, Phytokeys 12: 30. 2012.

Type: China, Hainan *Pénicaud 43* (lectotype P).

Distribution in India: The range of this species is unclear.

Distribution outside India: China, Cambodia, Indonesia, Laos, Peninsular Malaysia, Myanmar, Philippines, Sri Lanka, Thailand, Vietnam (fide Lu et al. 2011).

Image: Some of the type specimens can be found online.

GenBank: Sequences from Kocyan et al. (2007) and De Boer et al. (2012) from Chinese and Thai material, all under *Gymnopetalum scabrum*, e.g., HE661469., HE661297.

Comments: The synonymizations for the most part follow Lu et al. (2011), except for the recently published varieties whose status needs further evaluation.

**84.** ***Trichosanthes tricuspidata*** Lour., Fl. Cochinch. 2: 589. 1790.

Type: Vietnam, *Loureiro* s.n. (not at BM fide John Hunnex, 23 Aug 2012; herbarium ?).

*Trichosanthes tricuspidata* Lour. var. *strigosa* Mitra & Bandyop., J. Bombay. Nat. Hist. Soc. 96(2): 374. 1998.

Type: India, West Bengal, Coochbehar (Jamalda), 22 Aug. 1995, *S. Bandyopadhyay 2904* (not seen).

*Trichosanthes palmata* Roxb., Fl. Ind. 3: 704. 1832, non L., 1753, nom. illeg.

*Trichosanthes burmensis* Kundu var. *alba* Kundu, J. Bombay. Nat. Hist. Soc. 43(3): 382. 1942. Type: Upper Myanmar, Maymyo, July 1888, *Badul Khan* (*King’s Collector*) 130 (CAL).

Distribution in India: West Bengal?

Distribution outside India: Myanmar, Thailand, Vietnam.

Image: http://www.flowersofindia.net/catalog/slides/Indrayan.html

GenBank: Sequences from De Boer et al. (2012) from two Thai specimens that appear to represent different species: HE661459, HE661544.

Comments: Duyfjes and Pruesapan (2004) doubt the occurrence of *Trichosanthes tricuspidata* in India. According to them, the species only occurs in Myanmar, Thailand and Vietnam, West Malaysia, and east to the Moluccas. Fide Hugo De Boer (pers. comm. 24 Oct. 2012), the type of *Trichosanthes burmensis* var. *alba* Kundu resembles material of *Trichosanthes tricuspidata* subsp. *tricuspidata* from Thailand. The collection label states that the flowers were white, which also matches *T. tricuspidata*.

**85. *Trichosanthes truncata*** C.B. Clarke, Fl. Brit. India 2(6): 608. 1879.

Syntypes: India, Meghalaya, alt. 1000 ft, *J.D. Hooker*s.n. Khasia Hills, alt. 4000 ft, (Cherra Coal-pit), *J.D. Hooker & Thomson*s.n., 1188 (CAL photo available from SSR, K), Darjeeling, 10 March 1871, *C.B. Clarke 13973B* (CAL photo available from SSR, K).

*Trichosanthes ovata* Cogn. in A. & C. DC., Monogr. Phan. 3: 365. 1881.

Type: India, Sikkim, *Thomson* s.n. (L, LE).

Distribution in India: Andhra Pradesh, Arunachal Pradesh, Assam, Meghalaya, Sikkim, West Bengal.

Distribution outside India: Bangladesh, Bhutan, China, Thailand, Vietnam.

Image: Nothing reliable found online.

GenBank: Sequences from De Boer et al. (2012), e.g., HE661547, HE661461.

Comments: Further synonymous names listed by Lu et al. (2011).

Comments: The synonymization of *Trichosanthes ovata* follows Jeffrey (1980, 1982).

**86. *Trichosanthes tubiflora*** (Wight & Arn.) H.J.De Boer, Phytokeys 12: 29. 2012. *Bryonia tubiflora* Wight & Arn., Prodr. Fl. Ind. Orient. 1: 347. 1834.

*Gymnopetalum tubiflorum* (Wight & Arn.) Cogn. in A. & C. DC., Monogr. Phan. 3: 388. 1881.

Type: Sri Lanka, Trincomalee, 1 Feb. 1796, *Rottler* s.n. ex Herb. Klein in Herb. Wight Cat. 1118 (K, E).

*Gymnopetalum wightii* Arn., Madras J. Lit. Sci. 12: 52. 1840 and J. Bot. 3: 278. 1841.

Type: Sri Lanka, *Wight 1146* (K).

Distribution in India: Kerala.

Distribution outside India: Sri Lanka.

Image: A photo of the flowers is included in De Boer et al. (2010).

GenBank: No published sequences available.

Comments: Based on molecular data, this is close to *Trichosanthes dioica*, not the other species formerly placed in the genus *Gymnopetalum*.

**87. *Trichosanthes villosula*** Cogn. in A. & C. DC., Monogr. Phan. 3: 362. 1881.

Type: India, Tamilnadu, near Mt. Nilgiri, *Hohenacker 1507* (G-BOISS, P, K).

*Trichosanthes villosula* Cogn. var. *nilgirrensis* Kundu, J. Bombay Nat. Hist. Soc. 43(3): 375. 1942.

Type: India, Kerala, Coonoor, Nilgiris, alt. 6000 ft., Nov. 1884, *J.S. Gamble 15733* (CAL photo available from SSR).

Distribution in India: Andhra Pradesh, Assam, Karnataka, Kerala, Nagaland, Tamil Nadu, West Bengal.

Distribution outside India: Banagladesh, China.

Image: Nothing reliable found online.

GenBank: No published sequences available.

Comments: Jeffrey (1980) placed *Trichosanthes villosula* under *Trichosanthes lobata*; we here follow the more recent treatment by Lu et al. (2011) in maintaining *Trichosanthes villosula* as separate.

**88. *Trichosanthes wallichiana*** (Ser.) Wight, Madras J. Lit. Sci. 12: 52. 1840.

*Involucraria wallichiana* Ser., Mém. Soc. Phys. Genéve 3(1): 25, 31. t. 5. 1825.

Type: Nepal, *Wallich* s.n. (G-DC).

*Trichosanthes palmata* L. var. *scotanthus* C.B. Clarke, Fl. Brit. India 2(6): 607. 1879, nom. nud.

*Trichosanthes bracteata* (Lam.) Voigt var. *scotanthus* (C.B. Clarke) Handel-Mazzetti, Symb. Sin. 7(4): 1065. 1936.

Type: Eastern India, *Sonnerat* s.n. (P).

Distribution in India: Arunachal Pradesh, Assam, Bihar, Himachal Pradesh, Manipur, Meghalaya, Nagaland, Sikkim, Tripura, West Bengal

Distribution outside India: Nepal, China (Guangdong, Guangxi, Guizhou, Xizang, Yunnan).

Image: Nothing reliable found online.

GenBank: No published sequences available.

Comments: Chakravarty (1982) writes that *Trichosanthes wallichiana* is “very closely allied to *Trichosanthes bracteata* (Lam.) Voigt from which it can be separated by the membranous leaves with black-dotted glands at the base.”

**89. *Zanonia indica*** L., Sp. Pl., ed. 2. 2: 1457. 1763.

Type: India, Kerala, Malabaria. Lectotype: “Penar-valli mas” in Rheede, Hort. Malab. 8: 39. t. 49, 1688, designated by Keraudren-Aymonin in Aubréville & Leroy (ed.), Fl. Cambodge Laos Viêt-Nam 15: 18. 1975.

*Zanonia indica* L. var. *pubescens* Cogn. in A. & C. DC., Monogr. Phan. 3: 927. 1881.

Syntypes: India, Himalaya and East Bengal, *Griffith 2521* (K, P). Java, *Blume* s.n. (Herb. Lung. Bat., P). Borneo, *Korthals* s.n. (Herb. Lung. Bat.), Bangarmassing, *J. Motley 804 et 920* (K).

Distribution in India: Andaman and Nicobar Islands, Assam, Goa, Karnataka, Kerala, Maharashtra, Meghalaya, Sikkim, Tamil Nadu, West Bengal.

Distribution outside India: Sri Lanka, S China, Indochina, through Malesia east to New Guinea.

Image: See De Wilde and Duyfjes (2007a).

GenBank: Sequences from Schaefer et al. (2009), e.g., EU436396, EU436345.

Comments: De Wilde and Duyfjes (2007a) discuss the species’ unusual morphology.

**90. *Zehneria bodinieri*** (H. Lév.) W.J.de Wilde & Duyfjes, Thai Forest Bull., Bot. 32: 17. 2004.

*Melothria bodinieri* H. Lév., Fl. Kouy-Tchéou 112. 1914.

*Pilogyne bodinieri* (H. Lév.) W.J.de Wilde & Duyfjes, Reinwardtia 12(5): 410. 2009.

Lectotype designated by De Wilde and Duyfjes (2004b): China, Guangzhou, Kouyan, *Bodinier 1957* (E, P).

*Melothria perpusilla* (Blume) Cogn. var. *subtruncata* Cogn. in A. & C. DC., Monog. Phan. 3: 608. 1881.

Syntypes: India, *Wight 1151* (CAL image available from SSR; K, LE, W), Sri Lanka, *Thwaites 1613* (BR, CAL image available from SSR, G, K, LE, P).

Distribution in India: Karnataka, Kerala, Tamil Nadu, also North India.

Distribution outside India: China, Myanmar, Sri Lanka, Sumatra, Malaysia, Sabah also Thailand, Vietnam, Cambodia, Laos, Vietnam, Peninsular Malaysia, Philippines (Palawan)

Image: Nothing reliable found online.

GenBank: Sequences from Kocyan et al. (2007) and Schaefer and Renner (2011b), e.g., DQ536614, HQ202008.

Comments: The synonymization of *Melothria perpusilla* (Blume) Cogn. var. *subtruncata* Cogn. follows Wilde and Duyfjes (2006). Jeffrey (1980), on the other hand, considers this name a synonym of *Zehneria maysorensis*.

**91. *Zehneria hookeriana*** (Wight & Arn.)Arn., J. Bot. 275. 1841.

*Bryonia hookeriana* Wight & Arn., Prodr. Fl. Ind. Orient. 1: 345. 1834.

Type: South India, *Wight Cat. no. 1117* (K).

Distribution in India: South India, Tamil Nadu. **Endemic.**

Image: http://apps.kew.org/herbcat/getImage.do?imageBarcode=K000036887

GenBank: No published sequences available.

Comments: Endemic to India fide De Wilde and Duyfjes (2006).

**92. *Zehneria japonica*** (Thunb.) H.Y. Liu, Bull. Natl. Mus. Nat. Sci. (Taichung) 1: 40. 1989.

*Bryonia japonica* Thunb., Syst. Veg., ed. 14, 870. 1784.

*Melothria japonica* (Thunb.) Cogn. in A. & C. DC., Monogr. Phan. 3: 599. 1881.

*Neoachmandra japonica* (Thunb.) W.J. de Wilde & Duyfjes, Blumea 51(1): 22. 2006.

Type: Japan, Nagasaki, *Thunberg* (UPS-THUNB 22826).

*Neoachmandra indica* (Lour.) W.J. de Wilde & Duyfjes, Blumea 51(1): 21. 2006.

*Zehneria indica* (Lour.) Keraudren-Aymonin in Aubréville & Leroy (ed.), Fl. Cambodge Laos Viêt-Nam 15: 52. 1975.

*Aechmandra indica* (Lour.) Arn., Hook. Journ. Bot. 3: 274. 1841

*Melothria indica* Lour., Fl. Cochinch. 1: 35. 1790.

Type: Vietnam, Tourane, *Loureiro* s.n. (not found in BM fide J. Hunnex, 6 Sep. 2012, contra de Wilde and Duyfjes, Thai. Bull. 2004), neotype *Squires 14* (BM), designated by Jeffrey (1980).

*Bryonia leucocarpa* Blume, Bijdr. Fl. Ned. Ind. 15: 924. 1826.

*Melothria leucocarpa* (Blume) Cogn. in A. & C. DC., Monogr. Phan. 3: 601. 1881.

*Neoachmandra leucocarpa* (Blume) W.J.de Wilde & Duyfjes, Blumea 51(1): 23. 2006

Lectotype: Java, *Blume* s.n. (L, barcode L0130099).

*Melothria leucocarpa* (Blume) Cogn. var. *triloba* (C.B. Clarke) Chakrav., Chakrav., Rec. Bot. Surv. India 17(1): 154. 1959.

Lectotype: India, *Wallich Cat. No. 6707* (K-W).

*Melothria odorata* C.B. Clarke, Fl. Brit. India 2(6): 626. 1879.

*Neoachmandra odorata* (C.B. Clarke) W.J.de Wilde & Duyfjes, Blumea 51(1): 27. 2006.

Syntypes: India, *Hamilton* in *Wallich Cat. 6706A,B,C* (herbarium), as *Bryonia odorata* Buch.-Ham. Northwest Himalaya; *Royle* s.n. (herbarium), “Throughout the plain of East Bengal, common, and ascending the hills to 700ft alt.”

*Melothria odorata* C.B. Clarke var. *triloba* C.B. Clarke, Fl. Brit. India 2(6): 626. 1879.

*Melothria zehnerioides* Haines, J. Proc. Asiat. Soc. Bengal 15: 315. 1920.

Type: N India, *Haines 4510* (herbarium?).

Distribution in India: Arunachal Pradesh, Assam, Bihar, Himachal Pradesh, Meghalaya, Nagaland, Punjab, Sikkim, Uttar Pradesh, Uttarakhand, West Bengal.

Distribution outside India: Thailand, China, Japan; Indonesia (Java, Sumatra).

Image: Nothing reliable found online.

GenBank: Sequences from Kocyan et al. (2007), as *Neoachmandra japonica*, e.g., DQ536753, DQ648192.

Comments: De Wilde and Duyfjes (2006b) have a very different concept of *Zehneria japonica* than does Jeffrey (most recently in Lu et al. 2011, Flora of China). They consider *Neoachmandra indica*, *Neoachmandra leucocarpa*, and *Neoachmandra odorata* separate species. They also provide a key and color photos of many *Zehneria* species. Molecular-phylogenetic work is needed to resolve the status of these various entities.

**93. *Zehneria maysorensis*** (Wight & Arn.) Arn., J. Bot. 3: 275. 1841.

*Pilogyne maysorensis* (Wight & Arn.) W.J.de Wilde & Duyfjes, Reinwardtia 12(5): 410. 2009.

*Bryonia maysorensis* Wight & Arn., Prodr. Fl. Ind. Orient. 1: 345. 1834.

Lectotype: South India, *Wight 1116* (K, P), designated by de Wilde and Duyfjes (2006b).

*Zehneria maysorensis* (Wight & Arn.) Arn. var. *umbellata* (Chakrav.) Kumari, Fl. Tamil Nadu Ind., Ser. 1: 175. 1983.

*Melothria mucronata* (Blume) Cogn.var. *umbellata* Chakrav., Rec. Bot. Surv. India 17(1): 150. 1959.

Syntypes: Peninsular India, Lower Pulneys, 1600 m, Sep., *Rodriguez 1955* (CAL 2 sheets, photos available from SSR), *Wight* (CAL, photo available from SSR).

*Zehneria maysorensis* (Wight & Arn.) Arn. var. *oblonga* V.P.Prasad & M.Prasad J. Econ. Taxon. Bot. 17(2): 471. 1993.

Type: India, Kerala State, Idukki District, Lower camp to Kumily area, 26 Dec. 1974, *K. Vivekananthan 45710* (MH).

Distribution in India: Andhra Pradesh, Karnataka, Kerala, Maharashtra, Meghalaya, Tamil Nadu. **Endemic.**

Image: http://apps.kew.org/herbcat/getImage.do?imageBarcode=K000742778

GenBank: No published sequences available.

Comment: De Wilde and Duyfjes (2004b) suggest that *Zehneria mucronata* (Blume) Miq., which is based on a Blume collection from Java (L) and widespread in Southeast Asia and Malesia, may be the same as *Zehneria maysorensis*, in which case it would be the older name.

**94. *Zehneria thwaitesii*** (Schweinf.) C. Jeffrey, Kew Bull. 15: 371. 1962.

*Cucumella thwaitesii* (Schweinf.) M.R. Almeida, Fl. Maharashtra 2: 314. 1998.

*Melothria thwaitesii* Schweinf., Reliq. Kotschy. 44, t. 29. 1868.

Lectotype: Sri Lanka, *Thwaites CP 2581* (K, BM, P, W) designated by de Wilde and Duyfjes (2004b).

*Melothria zeylanica* C.B. Clarke in Hook. f., Fl. Brit. Ind. 2: 626 (1879), nom. inval.

*Bryonia deltoidea* Arn., Pugill.: 19 (1836), nom illeg., non Schumach., 1827 = *Melothria deltoidea* (Arn.) Thwaites, Enum.: 124 (1859) nom. illegit., non (Schumach.) Benth. 1849. = *Neoachmandra deltoidea* (Arn.) W.J.de Wilde & Duyfjes, Blumea 51(1): 18 (79). 2006. Material: Sri Lanka, *Walker 273* (K).

Distribution in India: Kerala.

Distribution outside India: Africa, Madagascar, Sri Lanka (Wilde and Duyfjes 2006).

Image: Nothing reliable found.

GenBank: Unpublished sequences from Asian material of *Neoachmandra deltoidea* (EF065485) and African material of *Zehneria thwaitesii* (AM981145).

Comment: C.B. Clarke (1879) applied the invalid name *Melothria zeylanica*to this species. De Wilde and Duyfjes (2006) treat *Zehneria thwaitesii* under *Neoachmandra deltoidea*. Molecular data are needed to clarify the genus boundaries of *Zehneria*.

## Misapplied names and species erroneously or doubtfully recorded from India:

***Bryonia dioica*** Jacq.

Comment: The distribution range given for *Bryonia dioica* by Chakravarty (1982) “Afganistan, Iran, Iraq, Tropical Africa, Syria, Palestine” is based on an exceedingly broad concept of this species. A narrower circumscription (Jeffrey, 1969) is supported by nuclear and plastid molecular data (Volz and Renner 2009). *Bryonia dioica* then occurs from Spain throughout Eurasia south to Algeria and Morocco, Sardinia, Corsica, and the Greek Peninsula and east to mid-Poland; a distribution map is shown in Volz and Renner (2009).

***Bryonia multiflora*** Boiss. & Heldr.

Comment: Listed by Chakravarty (1982) based on misidentification of *Bryonia monoica* (see under that species).

***Cayaponia laciniosa*** (L.) C. Jeffrey, Kew Bull. 15(3): 346. 1962.

*Bryonopsis laciniosa* (L.) Naudin, Ann. Sci. Nat., Bot. sér. 5: 6. 1866.

*Bryonia laciniosa* L., Sp. Pl. 2: 1013. 1753. Type: “Habitat in Zeylona.” (Country assignment in error). Lectotype: Cultivated in the Netherlands, Hartekamp in 1736–1737, Herb. Clifford: 452, *Bryonia 1* (BM-000647451), designated by Jeffrey (1962).

Comment from M. Nee, New York Botanical Garden, pers. comm. to S. Renner in 2010: In 1962, Jeffrey thought that *Cayaponia laciniosa* was the correct name for *Cayaponia racemosa* (Mill.) Cogn. By 1971, however, he decided that *Cayaponia laciniosa* was a local Jamaican endemic. The fullest description would be from Hort. Cliff. 452 based on living plants that Linnaeus saw; Linnaeus erroneously equated syntypes of this plant with literature of a different genus and species from Asia.

***Citrullus ecirrhosus*** Cogn., Verh. Bot. Vereins Prov. Brandenburg 30:151. 1888.

*Colocynthis ecirrhosus* (Cogn.) Chakrav., Science & Culture 15: 32. 1949.

This species is from Africa and not a synonym of *Benincasa fistulosa*.

***Hemsleya graciliflora*** (Harms) Cogn. in Engl. Pflanzenr. IV. 275, 1 (Heft 66): 24, f. 7A–H. 1916.

*Alsomitra graciliflora* Harms, Bot. Jahrb. Syst. 29(5): 602. 1901.

Syntypes: China, W Wenchuan, Niangtzuling, *BvR 3134, 3136*, Hubei, *Henry 4452*, Sichuan, Wenchuan, Niangziling; *von Rosthorn 3134, 3136* (B, destroyed in WWII)

Comment: Accepted for India by Chakravarty (1982), while Jeffrey (1980) states that records for India are based on misidentifications of *Gomphogyne macrocarpa* (*Hemsleya macrocarpa*).

***Hodgsonia macrocarpa*** (Blume) Cogn. in A. & C. DC., Monogr. Phan. 3: 349. 1881. *Trichosanthes macrocarpa* Blume, Bijdr. Fl. Ned. Ind. 15: 935. 1826.

Type: Java, Mt. Salak, *Blume* s.n. (L).

*Trichosanthes listeri* Chakrav., J. Bombay Nat. Hist. Soc. 50(4): 895, pl. 2. 1952.

Type: Bangladesh [Bengal], Chittagong Hill Tracts, Burkul, March 4, 1876, *Lister 349* as to the flowers (CAL photo available from SSR). As Jeffrey (1982) noted, the type is a mixed collection, the flowers coming from *Hodgsonia macrocarpa* (now *Hodgsonia heteroclita*), the shoot from *Thladiantha cordifolia*. Jeffrey designated the flowers as the lectotype.

Comment: *Hodgsonia* has two species, *Hodgsonia macrocarpa* in Java, and *Hodgsonia heteroclita* in Northeast India, Bhutan, South China, Myanmar, Laos, Cambodia, Vietnam, Thailand, and Peninsular Malaysia. Jeffrey’s (1980) and Chakravarty’s (1982) listing of this name for India is based on a broader species concept, in which *Hodgsonia heteroclita* was part of *Hodgsonia macrocarpa* (De Wilde and Duyfjes 2001).

*Trichosanthes thwaitesii* Cogn. in A. & C. DC., Monogr. Phan. 3: 387. 1881, nom. illegit.

***Zanonia heterosperma*** Wall., Pl. Asiat. Rar. 2: 29. 1831.

*Gomphogyne heterosperma* (Wall.) Kurz, J. Asiat. Soc. Bengal 46: 105. 1877.

*Alsomitra heterosperma* (Wall.) M.Roem., Syn. Monogr. 2: 118. 1846.

*Hemsleya heterosperma* (Wall.) C. Jeffrey, Kew Bull. 36: 739. 1982.

Type: Myanmar (Burma), Ava, Mt. Taong Daong, *Wallich 1038* (K-W 3728).

Comment: Listed by Jeffrey (1982) as occurring in India, but according to De Wilde et al. (2007), the species is restricted to East Myanmar and Thailand.

***Zehneria perpusilla*** (Blume) Bole & M.R. Almeida, J. Bombay Nat. Hist. Soc. 79(2): 315. 1983.

*Melothria perpusilla* (Blume) Cogn. in A. & C. DC., Monogr. Phan. 3: 607. 1881.

*Cucurbita perpusilla* Blume, Cat. Gew. Buitenzorg (Blume) 105. 1823.

Lectotype: Java, *Blume* s.n. (L, barcode L0048312).

Comment: According to De Wilde and Duyfjes (2006), this Javanese species does not occur in India.

***Zehneria wallichii*** (C.B. Clarke) C. Jeffrey, Kew Bull. 34(4): 802. 1980.

*Neoachmandra wallichii* (C.B. Clarke) W. J.de Wilde & Duyfjes, Blumea 51(1): 32. 2006.

*Melothria wallichii* C.B. Clarke, Fl. Brit. India 2: 626. 1879.

Type: Myanmar, Pyay (formerly Prome), *Wallich 6706D* (K-W).

This species, included in Jeffrey’s (1980) Indian checklist, was collected in the center of Myanmar and appears not to occur in India. Chakravarty (1982) does not mention the name.

## Appendix

**Names treated in this checklist**

Accepted names for species occurring in India are set in bold, species only mentioned in comments are shown in non-bold, and synonymized names in italics.

**Actinostemma tenerum** Griff.

*Aechmandra conocarpa* Dalzell & A.Gibson

*Aechmandra epigaea* (Rottler) Arn.

*Aechmandra indica* (Lour.) Arn.

*Aechmandra rostrata* (Rottler) Arn.

*Aechmandra velutina* Dalzell & A.Gibson

*Aechmandra zeylanica* Thwaites

*Alsomitra clavigera* (Wall.) M.Roem., nom. nud.

*Alsomitra graciliflora* Harms

*Alsomitra heterosperma* (Wall.) M.Roem.

*Alsomitra integrifoliola* (Cogniaux) Hayata

*Alsomitra pubigera* Prain

*Benincasa cerifera* Savi

**Benincasa fistulosa** (Stocks) H. Schaef. & S.S. Renner

**Benincasa hispida** (Thunb.) Cogn.

*Benincasa pruriens* (Parkinson) W.J.de Wilde & Duyfjes L. forma *hispida* (Thunb.) W.J.deWilde & Duyfjes

*Biswarea tonglensis* (C.B. Clarke) Cogn.

**Blastania cerasiformis** (Stocks) A. Meeuse

*Blastania fimbristipula* Kotschy & Peyr.

*Blastania garcinii* (Burm.f.) Cogn.

*Bryonia alceifolia* Willd.

*Bryonia amplexicaulis* Lam.

**Bryonia aspera** Steven ex Ledeb.

*Bryonia callosa* Rottler

*Bryonia cochinchinensis* Lour.

*Bryonia cordifolia* L.

*Bryonia cucumeroides* Ser.

*Bryonia deltoidea* Arn., nom. illeg.

Bryonia dioica Jacq.

*Bryonia epigaea* Rottler

*Bryonia fimbristipula* Fenzl ex Stocks, nom. inval.

*Bryonia garcinii* (Burm.f.) Willd.

*Bryonia grandis* L.

*Bryonia hookeriana* Wight & Arn.

*Bryonia japonica* Thunb.

*Bryonia laciniosa* L.

*Bryonia leiosperma* Wight & Arn.

*Bryonia leucocarpa* Blume

*Bryonia maysorensis* Wight & Arn.

**Bryonia monoica** Aitch. & Hemsl.

Bryonia multiflora Boiss. & Heldr.

*Bryonia napaulensis* Ser.

*Bryonia palmata* L.

*Bryonia pedunculosa* Ser.

*Bryonia rheedei* Blume

*Bryonia rostrata* Willd.

*Bryonia sagittata* Blume

*Bryonia scabrella* L.f.

*Bryonia tubiflora* Wight & Arn.

*Bryonia umbellata* Willd.

*Bryonopsis bennettii* Miq.

*Bryonopsis courtallensis* Arn.

*Bryonopsis laciniosa* (L.) Naudin

*Bryonopsis laciniosa* (L.) Naudin var. *walkeri* Chakrav.

*Cayaponia laciniosa* (L.) C. Jeffrey

*Cephalandra indica* Naudin var. *palmata* C.B. Clarke, nom. superfl.

*Cerasiocarpum bennettii* (Miq.) Cogn.

*Cerasiocarpum zeylanicum* (Thwaites) C.B. Clarke

**Citrullus colocynthis** (L.) Schrad.

*Citrullus fistulosus* Stocks

*Citrullus vulgaris* Schrad. ex Eckl. & Zeyh. var. *fistulosus* (Stocks) J. L. Stewart

**Citrullus lanatus** (Thunb.) Matsum. & Nakai

*Citrullus lanatus* (Thunb.) Matsum. & Nakai var. *fistulosus* (Steward) Babu, nom. inval.

*Citrullus lanatus* (Thunb.) Matsum. & Nakai var. *fistulosus* (Stocks) Chakrav., nom. illeg.

*Citrullus vulgaris* Schrad. ex Eckl. & Zeyh.

*Coccinia cordifolia* (L.) Cogn.

*Coccinia cordifolia* (L.) Cogn. var. *wightiana* (M.Roem.) Cogn.

**Coccinia grandis** (L.) Voigt

*Coccinia grandis* (L.) Voigt var. *wightiana* (M.Roem.) Greb.

*Coccinia indica* Wight & Arn., nom. superfl., nom. illeg.

*Coccinia palmata* M.Roem.

*Coccinia wightiana* M.Roem.

*Colocynthis citrullus* (L.) Kuntze var. *fistulosus* (Stocks) Chakrav.

*Colocynthis ecirrhosus* (Cogn.) Chakrav.

*Colocynthis vulgaris* Schrad.

**Corallocarpus conocarpus** (Dalzell & A.Gibson) Hook.f. ex C.B. Clarke

*Corallocarpus courbonii* (Naudin) Cogn.

**Corallocarpus epigaeus** (Rottler) Benth. & Hook.f. ex C.B. Clarke

*Corallocarpus gracilipes* (Naudin) Cogn.

*Corallocarpus palmatus* Cogn.

**Corallocarpus schimperi** (Naudin) Hook.f.

*Corallocarpus velutinus* (Dalzell & A.Gibson) Hook.f. ex C.B. Clarke

*Ctenolepis cerasiformis* (Stocks) Hook.f.,

*Ctenolepis garcinii* (Burm.f.) Benth. & Hook.f.

*Ctenolepis welwitschii* (Hook.f.) Jafri

*Cucumella ritchiei* (Chakrav.) C. Jeffrey

*Cucumella silentvalleyi* Manilal, T. Sabu & P. J. Mathew

*Cucumella thwaitesii* (Schweinf.) M.R. Almeida

*Cucumis acutangulus* L.

*Cucumis anguinus* L.

*Cucumis callosus* (Rottler) Cogn.

*Cucumis colocynthis* L.

*Cucumis hardwickii* Royle

**Cucumis hystrix** Chakrav.

**Cucumis indicus** Ghebretinsae & Thulin

*Cucumis integrifolius* Roxb.

**Cucumis javanicus** (Miq.) Ghebretinsae &Thulin

**Cucumis leiospermus** (Wight & Arn.) Ghebretinsae & Thulin

**Cucumis maderaspatanus** L.

**Cucumis melo** L.

*Cucumis melo* L. forma *agrestis* (Naudin) W.J.de Wilde & Duyfjes

*Cucumis melo* L. subsp. *agrestis* (Naudin) Pangalo

*Cucumis melo* L. var. *agrestis* Naudin

*Cucumis melo* L. var. *culta* Kurz

*Cucumis melo* L. var. *momordica* Duthie & Fullar

*Cucumis melo* L. var. *pubescens* (Willd.) Kurz

*Cucumis melo* L. var. *utilissima* Duthie & Fullar

*Cucumis momordica* Roxb.

*Cucumis muriculatus* Chakrav.

**Cucumis prophetarum** L.

*Cucumis pubescens* Willd.

**Cucumis ritchiei** (C.B. Clarke) Ghebretinsae & Thulin

**Cucumis sativus** L.

*Cucumis sativus* L. forma *hardwickii* (Royle) W.J.de Wilde & Duyfjes

*Cucumis sativus* L. var. *sikkimensis* Hook.f.

**Cucumis setosus** Cogn.

**Cucumis silentvalleyi** (Manilal, T. Sabu & P.J. Mathew) Ghebretinsae & Thulin

*Cucumis trigonus* Roxb.

**Cucurbita argyrosperma** C.Huber

*Cucurbita citrullus* L.

**Cucurbita ficifolia** Bouché

*Cucurbita hispida* Thunb.

*Cucurbita lagenaria* L.

**Cucurbita maxima** Duchesne

*Cucurbita maxima* Duchesne var. *badagarensis* Mudaliar

*Cucurbita mixta* Pangalo

**Cucurbita moschata** (Duchesne ex Lam.) Duchesne

**Cucurbita pepo** L.

*Cucurbita pepo* L. var. *melopepo* Alef.

*Cucurbita pepo* L. var. *moschata* Duchesne ex Lam.

*Cucurbita pepo* L. var. *ovigera* Alef.

*Cucurbita perpusilla* Blume

*Cucurbita siceraria* Molina

*Cucurbita umbellata* Willd.

**Cyclanthera pedata** (L.) Schrad.

**Dactyliandra welwitschii** Hook.f.

*Dicaelospermum ritchiei* C.B. Clarke

**Diplocyclos palmatus** (L.) C. Jeffrey

*Diplocyclos palmatus* (L.) C. Jeffrey var. *walkeri* (Chakrav.) Babu

*Edgaria darjeelingensis* C.B. Clarke

*Edgaria darjeelingensis* C.B. Clarke var. *clarkeana* S. N. Biswas

*Euonymus chinensis* Lour.

*Gomphogyne cirromitrata* W.J.de Wilde & Duyfjes

**Gomphogyne cissiformis** Griff.

*Gomphogyne cissiformis* Griff. forma *villosa* (Cogn.) Mizush.

*Gomphogyne cissiformis* Griff.var. *villosa* Cogn.

*Gomphogyne heterosperma* (Wall.) Kurz

*Gomphogyne macrocarpa* Cogn.

Gomphogyne nepalensis W.J.de Wilde & Duyfjes

*Gymnopetalum chinense* (Lour.) Merr.

*Gymnopetalum cochinchinense* (Lour.) Kurz

*Gymnopetalum heterophyllum* Kurz

*Gymnopetalum integrifolium* (Roxb.) Kurz

*Gymnopetalum integrifolium* (Roxb.) Kurz var. *pectinatum* W.J.de Wilde & Duyfjes

*Gymnopetalum penicaudii* Gagnep.

*Gymnopetalum quinquelobum* Miq.

*Gymnopetalum scabrum* (Lour.) W.J.de Wilde & Duyfjes

*Gymnopetalum scabrum* (Lour.) W.J.de Wilde & Duyfjes var. *pectinatum* (W.J. de Wilde& Duyfjes) W.J.de Wilde & Duyfjes

*Gymnopetalum scabrum* (Lour.) W.J.de Wilde & Duyfjes var. *penicaudii* (Gagnep.) W.J.de Wilde& Duyfjes

*Gymnopetalum tubiflorum* (Wight & Arn.) Cogn.

*Gymnopetalum wightii* Arn.

*Gynostemma integrifoliolum* Cogn.

*Gynostemma laxum* (Wall.) Cogn.

*Gynostemma pedatum* Blume

**Gynostemma pentaphyllum** (Thunb.) Makino

*Gynostemma pentaphyllum* (Thunb.) Makino forma *simplicifolium* (Blume) W.J.de Wilde & Duyfjes

*Gynostemma simplicifolium* Blume

*Hemsleya cirromitrata* (W.J.de Wilde & Duyfjes) H. Schaef. & S.S.Renner

*Hemsleya graciliflora* (Harms) Cogn.

*Hemsleya heterosperma* (Wall.) C. Jeffrey

**Hemsleya macrocarpa** (Cogn.) C. Y. Wu ex C. Jeffrey

*Hemsleya trifoliolata* Cogn.

*Herpetospermum caudigerum* Wall. ex C.B. Clarke, nom. illeg.

**Herpetospermum darjeelingense** (C.B. Clarke) H.Schaef. & S.S.Renner

**Herpetospermum pedunculosum** (Ser.) Baill.

**Herpetospermum tonglense** (C.B. Clarke) H. Schaef. & S.S. Renner

**Hodgsonia heteroclita** (Roxb.) Hook.f. & Thomson

**Hodgsonia macrocarpa** (Blume) Cogn.

**Indofevillea khasiana** Chatterjee

*Involucraria lepiniana* Naudin

*Involucraria wallichiana* Ser.

*Karivia amplexicaulis* (Lam.) Arn.

*Karivia javanica* Miq.

*Karivia rheedei* (Blume) M.Roem.

*Karivia umbellata* (Willd.) Arn.

*Kedrostis bennettii* (Miq.) W.J.de Wilde & Duyfjes

**Kedrostis courtallensis** (Arn.) C. Jeffrey

**Kedrostis foetidissima** (Jacq.) Cogn.

*Kedrostis rostrata* (Willd.) Cogn.

**Lagenaria siceraria** (Molina) Standl.

**Luffa acutangula** (L.) Roxb.

*Luffa acutangula* (L.) Roxb. var. *amara* (Roxb.) C.B. Clarke

*Luffa acutangula* (L.) Roxb. forma *amara* (Roxb.) W.J.de Wilde & Duyfjes

*Luffa aegyptiaca* Mill.

*Luffa aegyptiaca* Mill. forma *sylvestris* (Miq.) W.J.de Wilde & Duyfjes

*Luffa amara* Roxb.

*Luffa cordifolia* Blume

**Luffa cylindrica** (L.) M.Roem.

*Luffa cylindrica* (L.) M.Roem. var. *minor* Chakrav., nom. nud.

**Luffa echinata** Roxb.

*Luffa echinata* Roxb.var. *longistyla* C.B. Clarke

**Luffa graveolens** Roxb.

*Luffa hermaphrodita* Singh & Bhandari

*Luffa kleinii* Wight & Arn.

*Luffa sylvestris* Miq.

*Luffa tuberosa* Roxb.

*Luffa umbellata* (Willd.) M.Roem.

*Melothria amplexicaulis* (Lam.) Cogn.

*Melothria angulata* Chakrav.

*Melothria assamica* Chakrav.

*Melothria assamica* Chakrav. var. *scabra* Chakrav.

*Melothria bicirrhosa* C.B. Clarke

*Melothria bodinieri* H. Lév.

*Melothria deltoidea* (Arn.) Thwaites, nom. illeg.

*Melothria fimbristipula* (Kotschy & Peyr.) G. Roberty

*Melothria heterophylla* (Lour.) Cogn.

*Melothria indica* Lour.

*Melothria japonica* (Thunb.) Cogn.

*Melothria javanica* (Miq.) Panigrahi & Misra

*Melothria javanica* (Miq.) Panigrahi & Misra var. *scabra* (Chakrav.) Naithani

*Melothria leiosperma* (Wight & Arn.) Cogn.

*Melothria leucocarpa* (Blume) Cogn.

*Melothria leucocarpa* (Blume) Cogn. var. *triloba* (C.B. Clarke) Chakrav.

*Melothria maderaspatana* (L.) Cogn.

*Melothria mucronata* (Blume) Cogn. var. *umbellata* Chakrav.

*Melothria odorata* C.B. Clarke

*Melothria odorata* C.B. Clarke var. *triloba* C.B. Clarke

*Melothria ovata* Cogn.

*Melothria perpusilla* (Blume) Cogn.

*Melothria perpusilla* (Blume) Cogn. var. *subtruncata* Cogn.

*Melothria ritchiei* Chakrav.

*Melothria thwaitesii* Schweinf.

*Melothria wallichii* C.B. Clarke

*Melothria zehnerioides* Haines

*Melothria zeylanica* Koen. ex Wight & Arn., nom. inval.

*Modecca bracteata* Lam.

**Momordica balsamina** L.

*Momordica calcarata* Colebr. ex Wall., nom. nud.

**Momordica charantia** L.

*Momordica charantia* L. var. *muricata* (Willd.) Chakrav.

**Momordica cochinchinensis** (Lour.) Spreng.

*Momordica cylindrica* L.

**Momordica cymbalaria** Fenzl ex Naudin

**Momordica denudata** (Thwaites) C.B. Clarke

**Momordica dioica** Roxb. ex Willd.

*Momordica dioica* Roxb. ex Willd. var. *denudata* Thwaites

*Momordica lanata* Thunb.

*Momordica macrophylla* Gage

*Momordica muricata* Willd.

*Momordica pedata* L.

*Momordica renigera* Wall. ex G. Don

**Momordica sahyadrica** Kattuk. & V.T.Antony

**Momordica subangulata** Blume

*Momordica subangulata* Blume subsp. *renigera* (Wall. ex G. Don) W.J.de Wilde

*Momordica tuberosa* (Roxb.) Cogn.

*Momordica tubiflora* Roxb.

*Momordica umbellata* (Willd.) Roxb.

*Mukia javanica* (Miq.) C. Jeffrey

*Mukia leiosperma* (Wight & Arn.) Arn.

*Mukia maderaspatana* (L.) M.Roem.

*Mukia ritchiei* (C.B. Clarke) W.J.de Wilde & Duyfjes

*Mukia scabrella* (L.f.) Arn.

*Muricia cochinchinensis* Lour.

*Neoachmandra deltoidea* (Arn.) W.J.de Wilde & Duyfjes

*Neoachmandra indica* (Lour.) W.J.de Wilde & Duyfjes

*Neoachmandra japonica* (Thunb.) W.J.de Wilde & Duyfjes

*Neoachmandra leucocarpa* (Blume) W.J.de Wilde & Duyfjes

*Neoachmandra odorata* (C.B. Clarke) W.J.de Wilde & Duyfjes

*Neoachmandra wallichii* (C.B. Clarke) W.J.de Wilde & Duyfjes

**Neoalsomitra clavigera** (Wall.) Hutch.

*Neoalsomitra clavigera* (Wall.) Hutch.var. *hookeri* (C.B. Clarke) Chakrav.

*Neoalsomitra integrifoliola* (Cogniaux) Hutchinson

*Neoluffa sikkimensis* Chakrav.

*Pilogyne bodinieri* (H. Lév.) W.J.de Wilde & Duyfjes

*Pilogyne maysorensis* (Wight & Arn.) W.J.de Wilde & Duyfjes

*Praecitrullus fistulosus* (Stocks) Pangalo

*Rhynchocarpa epigaea* (Rottler) Naudin

*Rhynchocarpa epigaea* (Rottler) Naudin var. *gracilipes* Naudin

*Rhynchocarpa schimperi* Naudin

*Rynchocarpa rostrata* (Rottler) Naudin

**Schizopepon bicirrhosus** (C.B. Clarke) C. Jeffrey

**Schizopepon longipes** Gagnep.

**Schizopepon macranthus** Handel-Mazzetti

*Schizopepon wardii* Chakrav.

*Scotanthus tubiflorus* (Roxb.) Naudin

*Sechium americanum* Poir.

*Sechium edule* (Jacq.) Sw.

**Siraitia sikkimensis** (Chakrav.) C. Jeffrey

**Sicyos edulis** Jacq.

*Sicyos garcinii* Burm.f.

**Solena amplexicaulis** (Lam.) Gandhi

*Solena angulata* (Chakrav.) Babu

**Solena heterophylla** Lour. subsp. **heterophylla**

**Solena heterophylla** Lour. subsp. **napaulensis** (Ser.) W.J.de Wilde &Duyfjes

**Solena umbellata** (Willd.) W.J. de Wilde & Duyfjes

*Thladiantha calcarata* C.B. Clarke var. *subglabra* Cogn.

*Thladiantha calcarata* C.B. Clarke, nom. nud.

**Thladiantha cordifolia** (Blume) Cogn.

*Thladiantha heptadactyla* Cogn.

**Thladiantha hookeri** C.B. Clarke

*Thladiantha hookeri* C.B. Clarke var. *irregularis* Chakrav.

*Thladiantha hookeri* C.B. Clarke var. *palmatifolia* Chakrav.

*Thladiantha hookeri* C.B. Clarke forma *trifoliolata* (Cogn.) Chakrav.

*Thladiantha pentadactyla* Cogn.

*Trichosanthes anguina* L.

**Trichosanthes anaimalaiensis** Bedd.

**Trichosanthes bracteata** (Lam.) Voigt

*Trichosanthes bracteata* (Lam.) Voigt var. *scotanthus* (C.B. Clarke) Handel-Mazzetti

*Trichosanthes bracteata* (Lam.) Voigt var. *tomentosa* (C.B. Clarke) Chakrav., nom. illeg.

*Trichosanthes brevibracteata* Kundu

*Trichosanthes brevibracteata* Kundu var. *longirostrata* Kundu

*Trichosanthes brevibracteata* Kundu var. *sublobata* Kundu

*Trichosanthes burmensis* Kundu

*Trichosanthes burmensis* Kundu var. *alba* Kundu

*Trichosanthes chinensis* Ser.

**Trichosanthes cordata** Roxb.

**Trichosanthes costata** Bl.

**Trichosanthes cucumerina** L.

**Trichosanthes cucumeroides** (Ser.)Maxim.

*Trichosanthes cucumeroides* (Ser.)Maxim. var. *dicaelosperma* (C.B. Clarke) S. K. Chen

*Trichosanthes cuspidata* Lam.

*Trichosanthes dicaelosperma* C.B. Clarke

**Trichosanthes dioica** Roxb.

*Trichosanthes dioica* Roxb. var. *sagittifolia* Chakrav.

**Trichosanthes dunniana** H. Lév.

*Trichosanthes foetidissima* Jacq.

*Trichosanthes heteroclita* Roxb.

*Trichosanthes himalensis* C.B. Clarke

*Trichosanthes himalensis* C.B. Clarke var. *glabrior* C.B. Clarke

*Trichosanthes himalensis* C.B. Clarke var. *himalensis*

*Trichosanthes himalensis* C.B. Clarke var. *indivisa* Chakrav., nom. illeg.

*Trichosanthes himalensis* C.B. Clarke var. *sikkimensis* (Kundu) Thoth.

*Trichosanthes horsfieldii* Miq.

*Trichosanthes integrifolia* (Roxb.) Kurz

**Trichosanthes kerrii** Craib

**Trichosanthes khasiana** Kundu

**Trichosanthes lepiniana** (Naudin) Cogn.

*Trichosanthes listeri* Chakrav.

**Trichosanthes lobata** Roxb.

*Trichosanthes macrocarpa* Blume

*Trichosanthes macrosiphon* Kurz

*Trichosanthes majuscula* (C.B. Clarke) Kundu

*Trichosanthes multiloba* Miq. var. *majuscula* C.B. Clarke

**Trichosanthes nervifolia** L.

*Trichosanthes ovata* Cogn.

**Trichosanthes ovigera** Blume

*Trichosanthes ovigera* Blume var. *sikkimensis* Kundu

*Trichosanthes ovigera* Blume subsp. *cucumeroides* (Ser.) C. Jeffrey

*Trichosanthes pachyrrhachis* Kundu

*Trichosanthes palmata* L. var. *scotanthus* C.B. Clarke, nom. nud.

*Trichosanthes palmata* L. var. *tomentosa* Heyne ex C.B. Clarke

*Trichosanthes palmata* Roxb., nom. illeg.

*Trichosanthes perrottetiana* Cogn.

Trichosanthes pilosa Lour.

*Trichosanthes prazeri* Kundu

*Trichosanthes pubera* Blume subsp. *rubriflos* (Thorel ex Cayla) Duyfjes & Pruesapan

**Trichosanthes quinquangulata** A. Gray

**Trichosanthes rubriflos** Thorel ex Cayla

**Trichosanthes scabra** Lour.

*Trichosanthes scabra* Lour. var. *pectinata* (W.J.de Wilde & Duyfjes) H.J.De Boer

*Trichosanthes scabra* Lour. var. *penicaudii* (Gagnep.) H.J.De Boer

*Trichosanthes thwaitesii* Cogn.

*Trichosanthes tomentosa* Chakrav.

**Trichosanthes tricuspidata** Lour.

*Trichosanthes tricuspidata* Lour. var. *strigosa* Mitra & Bandyop.

**Trichosanthes truncata** C.B. Clarke

**Trichosanthes tubiflora** (Wight & Arn.) H.J.De Boer

**Trichosanthes villosula** Cogn.

*Trichosanthes villosula* Cogn. var. *nilgirrensis* Kundu

**Trichosanthes wallichiana** (Ser.) Wight

*Trichosanthes wallichiana* (Ser.) Wight var. *majuscula* (C.B. Clarke) Cogn.

*Tripodanthera cochinchinensis* (Lour.) M.Roem.

*Vitis pentaphylla* Thunb.

*Warea tonglensis* C.B. Clarke

*Zanonia clavigera* Wall.

Zanonia heterosperma Wall.

**Zanonia indica** L.

*Zanonia indica* L. var. *pubescens* Cogn.

*Zanonia laxa* Wall.

*Zehneria angulata* (Chakrav.) J. L. Ellis

**Zehneria bodinieri** (H. Lév.) W.J.de Wilde & Duyfjes

*Zehneria cerasiformis* Stocks

**Zehneria hookeriana** (Wight & Arn.)Arn.

*Zehneria indica* (Lour.) Keraudren-Aymonin

**Zehneria japonica** (Thunb.) H.Y. Liu

**Zehneria maysorensis** (Wight & Arn.) Arn.

*Zehneria maysorensis* (Wight & Arn.) Arn. var. *oblonga* V.P.Prasad & M.Prasad

*Zehneria maysorensis* (Wight & Arn.) Arn. var. *umbellata* (Chakrav.) Kumari

Zehneria mucronata (Blume) Miq.

*Zehneria perpusilla* (Blume) Bole & M.R. Almeida

**Zehneria thwaitesii** (Schweinf.) C. Jeffrey

*Zehneria umbellata* (Klein ex Willd.) Thwaites

*Zehneria umbellata* (Klein ex Willd.) Thwaites var. *napaulensis* (Ser.) C.B. Clarke

*Zehneria wallichii* (C.B. Clarke) C. Jeffrey

## References

[B1] Ali Khan A (2002)*Actinostemma tenerum* Griff., Cucurbitaceae, a new phytogeographic record from Aligarh, Uttar Pradesh. J. Bombay Nat. Hist. Soc. 99. (2): 365–366.

[B2] Ali MA Pandey AK Lee J (2009) Taxonomic relationships among the genera of subfamily Cucurbitoideae (family Cucurbitaceae) from India inferred from ITS sequences of nuclear ribosomal DNA. Phytomorphology 59(3&4): 127–140.

[B3] Bhandari MM Singh D (1964) *Dactyliandra* (Hook.f.) Hook. F.: A cucurbitaceous genus new to the Indian flora. Kew Bulletin 19 (1): 133-138 doi: 10.2307/4108301

[B4] Chakravarty HL (1946) Studies in Indian Cucurbitaceae with special remarks on distribution and uses of economic species. Indian Journal of Agricultural Science 16: 47-50

[B5] Chakravarty HL (1959) Monograph of Indian Cucurbitaceae (taxonomy and distribution). Records of the Botanical Survey of India 17: 1-234

[B6] Chakravarty HL (1982)*Cucurbitaceae in* SK Jain (ed.) *Fascicles of Flora of India*, 11, Botanical Survey of India, Calcutta.

[B7] Chopra RN Nayar SC Chopra IC (1956)*Glossary of Indian Medicinal Plants*. Council of scientific and industrial research, New Delhi, India.

[B8] Clarke CB (1879) Cucurbitaceae. In Hooker JD, Flora of British India 2: 604-635

[B9] Dane F Lang P (2004) Sequence variation at cpDNA regions of watermelon and related wild species: implications for the evolution of *Citrullus* haplotypes. American Journal of Botany 91 (11): 1922-1929 doi: 10.3732/ajb.91.11.1922 21652338 10.3732/ajb.91.11.1922

[B10] De Boer HJ Thulin M (2012) Synopsis of *Trichosanthes* (Cucurbitaceae) based on recent molecular phylogenetic data. PhytoKeys 12: 23-33 doi: 10.3897/phytokeys.12.2952 22645411 10.3897/phytokeys.12.2952 PMC3349053

[B11] De Boer HJ Schaefer H Thulin M Renner SS (2012) Evolution and loss of long-fringed petals: A case study using a dated phylogeny of the snake gourds, *Trichosanthes* (Cucurbitaceae). BMC Evolutionary Biology 12: 108. doi: 10.1186/1471-2148-12-108 10.1186/1471-2148-12-108 PMC3502538 22759528

[B12] Decker-Walters DS Sang-Min Chung I Staub JE (2004) Plastid sequence evolution: A new pattern of nucleotide substitutions in the Cucurbitaceae. Journal of Molecular Evolution 58: 606-614 doi: 10.1007/s00239-004-2585-z 15170263 10.1007/s00239-004-2585-z

[B13] De Wilde WJJO Duyfjes BEE (2001) Taxonomy of *Hodgsonia* (Cucurbitaceae), with a note on the ovules and seeds. Blumea 46: 169-179

[B14] De Wilde WJJO Duyfjes BEE (2002) Synopsis of *Momordica* (Cucurbitaceae) in SE Asia and Malesia, Bot. Zhurna 87, 3: 142.

[B15] De Wilde WJJO Duyfjes BEE (2003) Revision of *Neoalsomitra* (Cucurbitaceae). Blumea 48: 99-121

[B16] De Wilde WJJO Duyfjes BEE (2004a) *Kedrostis* Medik. (Cucurbitaceae) in Asia. Reinwardtia 12: 129-133

[B17] De Wilde WJJO Duyfjes BEE (2004b) *Zehneria* (Cucurbitaceae) in Thailand, with a note on the Indian *Zehneria maysorensis*. Thai Forest Bulletin, Botany 32: 15-31

[B18] De Wilde WJJO Duyfjes BEE (2004c) Review of the genus *Solena* (Cucubritaceae). Blumea 49: 69-81 doi: 10.3767/000651904X486197

[B19] De Wilde WJJO Duyfjes BEE (2006a) *Mukia* Arn. (Cucurbitaceae) in Asia, in particular in Thailand. Thai Forest Bulletin, Botany 34: 38-52

[B20] De Wilde WJJO Duyfjes BEE (2006b) Redefinition of *Zehneria* and four new related genera (Cucurbitaceae), with an enumeration of the Australasian and Pacific species. Blumea 51: 1-88 doi: 10.3767/000651906X622346

[B21] De Wilde WJJO Duyfjes BEE (2006c) Review of the genus *Gymnopetalum* (Cucurbitaceae) Blumea 51: 281–296. doi: 10.3767/000651906X622229

[B22] De Wilde WJJO Duyfjes BEE (2007a) Diversity in *Zanonia indica* (Cucurbitaceae). Blumea 52: 281-290 doi: 10.3767/000651907X609016

[B23] De Wilde WJJO Duyfjes BEE (2007b) The wild species of *Cucumis* L. (Cucurbitaceae) in South-East Asia. Adansonia, sér. 3, 29: 239–248.

[B24] De Wilde WJJO Duyfjes BEE (2008a) Cucurbitaceae. *Flora of Thailand* 9, 4: 135 pp., 12 plates. Niran Hetrakul, Prachachon Co. Ltd., Thailand.

[B25] De Wilde WJJO Duyfjes BEE (2010) Cucurbitaceae in Flora Malesiana, vol. 19. Leiden: Nationaal Herbarium Nederland.

[B26] De Wilde WJJO Duyfjes BEE (2008b) Miscellaneous South East Asian Cucurbit news. Reinwardtia 12: 267-274

[B27] De Wilde WJJO Duyfjes BEE van der Ham RWJM (2007) Revision of the genus *Gomphogyne* (Cucurbitaceae). Thai Forest Bulletin, Botany 35: 45-68

[B28] Duyfjes BEE Pruesapan K (2004) The genus *Trichosanthes* L. (Cucurbitaceae) in Thailand. Thai Forest Bulletin, Botany 32: 76-109

[B29] Garcia-Mas J and 34 coauthors (2012) The genome of melon (*Cucumis melo* L.). Proceedings of the National Academy of Science, USA 1009 (29): 11872-11877 10.1073/pnas.1205415109 PMC3406823 22753475

[B30] Holstein N (in press) Monograph of *Coccinia* (Cucurbitaceae). PhytoKeys10.3897/phytokeys.54.3285 PMC4547038 26312043

[B31] Holstein N Renner SS (2011) A dated phylogeny and collection records reveal repeated biome shifts in the African genus *Coccinia* (Cucurbitaceae). BMC Evolutionary Biology 11: 28. doi: 10.1186/1471-2148-11-28 10.1186/1471-2148-11-28 PMC3041684 21269492

[B32] Huang S and 87 coauthors (2009) The genome of the cucumber, *Cucumis sativus* L. Nature Genetics 41: 1275-1281 doi: 10.1038/ng.475 19881527 10.1038/ng.475

[B33] Jarvis C (2007)*Order out of Chaos: Linnaean Plant Names and their Types*. London: Linnean Society of London and the Natural History Museum, UK. Pp. 1016.

[B34] Jeffrey C (1962) Notes on Cucurbitaceae, including a proposed new classification of the family. Kew Bulletin 15 (3): 337-371 doi: 10.2307/4115586

[B35] Jeffrey C (1967) Cucurbitaceae. In: Milne-Redhead E, Polhill RM (Eds), Flora of Tropical East Africa.

[B36] Jeffrey C (1969) A review of the genus *Bryonia* (Cucurbitaceae). Kew Bulletin 23: 441-461 doi: 10.2307/4117182

[B37] Jeffrey C (1971) Further notes on Cucurbitaceae: II. The tribe Cucurbiteae. Kew Bulletin 25: 191-236 doi: 10.2307/4103207

[B38] Jeffrey C (1980) Further notes on Cucurbitaceae: V. The Cucurbitaceae of the Indian subcontinent. Kew Bulletin 34: 789-809 doi: 10.2307/4119071

[B39] Jeffrey C (1981) Further notes on Cucurbitaceae: VI. Cucurbitaceae of the Indian subcontinent: corrigenda and addenda. Kew Bulletin 36: 737-740 doi: 10.2307/4117916

[B40] Joseph JK Antony VT (2010) A taxonomic revision of the genus *Momordica* L. (Cucurbitaceae) in India. Indian Journal of Plant Genetic Resources 23 (2): 172-184

[B41] Khatoon S (2006) *Dactyliandra* (Hook.f.) Hook.f. (Cucurbitaceae) - a new generic record from Pakistan. International Journal of Biology and Biotechnology 3: 819-820

[B42] Kattukunnel JJ . see Joseph John.

[B43] Kocyan A Zhang LB Schaefer H Renner SS (2007) A multi-locus chloroplast phylogeny for the Cucurbitaceae and its implications for character evolution and classification. Molecular Phylogenetics and Evolution 44: 553-577 doi: 10.1016/j.ympev.2006.12.022 17321763 10.1016/j.ympev.2006.12.022

[B44] Kumari A Komal R Rajesh R Pandey AK (2009) *In Vitro* pollen germination, pollen tube growth and pollen viability in *Trichosanthes dioica* Roxb. (Cucurbitaceae). The International Journal of Plant Reproductive Biology 1 (2): 147-151

[B45] Li DZ Gao LM Li HT Wang H Ge XJ Liu JQ Chen ZD Zhou SL Chen SL Yang JB Fu CX Zeng CX Yan HF Zhu YJ Sun YS Chen SY Zhao L Wang K Yang T Duan GW (2011) Comparative analysis of a large dataset indicates that internal transcribed spacer (ITS) should be incorporated into the core barcode for seed plants. Proceedings of the National Academy of Science, USA 108 (49): 19641-19646 doi: 10.1073/pnas.1104551108 10.1073/pnas.1104551108 PMC3241788 22100737

[B46] Li HT Yang JB Li DZ Moller M Shah A (2010) A molecular phylogenetic study of *Hemsleya* (Cucurbitaceae) based on ITS, rpl16, trnH-psbA, and trnL DNA sequences. Plant Systematics and Evolution 285(1–2)): 23-32 doi: 10.1007/s00606-009-0252-y

[B47] Liao PC Tsai CC Chou CH Chiang YC (2012) Introgression between cultivars and wild populations of *Momordica charantia* L. (Cucurbitaceae) in Taiwan. International Journal of Molecular Science 13 (5): 6469-6491 doi: 10.3390/ijms13056469 10.3390/ijms13056469 PMC3382758 22754378

[B48] Lokesha R Vasudeva R (2001) Differential influence of pollen and stylar genotypes on lifespan of pistillate flowers in a monoecious herb, *Momordica tuberosa* (Cogn.) Roxb. (Cucurbitaceae). Current Science 81: 1628-1633

[B49] Lu A Huang L Chen SK Jeffrey C (2011) Cucurbitaceae. In: Wu ZY, Raven PH, Hong DY (Eds) *Flora of China*. Vol. 19. Missouri Botanical Garden Press, St. Louis.

[B50] Nadkarni KM Nadkarni AK (1976) Dr. K.M. Nadkarni’s Indian materia medica with Ayurvedic, Unani-tibbi, Siddha, allopathic, homeopathic, naturopathic & home remedies, appendices & indexes 1. Popular Prakasham, Mumbai, LXXII, 1319 pp.

[B51] Naithani HB (1990) Flowering plants of India, Nepal & Bhutan (not recorded in Sir J.D. Hooker’s Flora of British India). 711 pp. Dehra Dun (Surya Publications).

[B52] Nicolson DH Fosberg FR (2003)*The Forsters and the botany of the Second Cook Expedition* International Association for Plant Taxonomy. 760 pages.

[B53] Nicolson DH Suresh CR Manilal KS (1988)*An interpretation of Van Rheede’s Hortus malabaricus*. Regnum Vegetabile 119.

[B54] Pandey AK Anupama J Pujari MM (1997) Floral biology of *Trichosanthes dioica* Roxb. (Cucurbitaceae). In: Khan IA (Ed) *Frontiers in Plant Science*, pp. 937–945. The Book Syndicate, Hyderabad, India.

[B55] Pandey AK Jha A Komal R (2003) Development of female gametophyte and seed in *Trichosanthes dioica* Roxb. (Cucurbitaceae). In: Pandey AK, Dhakal MR (Eds) *Advances in Plant Reproductive Biology*. Vol. II, pp. 141–150. Narendra Publishing House, Delhi, India.

[B56] Pandey AK Varma SK Ali MA (2006) The genus *Luffa* in India: Diversity and conservation. In: Trivedi PC (Ed), *Global Biodiversity: Status and Conservation*, pp. 260–270. Pointer Publishers, Jaipur, India.

[B57] Parvathi S Kumar VJF (2002) Studies on chemical composition and utilization of the wild edible vegetable athalakkai (*Momordica tuberosa*). Plant Foods for Human Nutrition 57 (3/4): 215–222. doi: 10.1023/A:1021884406024 10.1023/a:1021884406024 12602930

[B58] Ramachandran K Subramaniam B (1983) Scarlet gourd, *Coccinia grandis*, little-known tropical drug plant. Economic Botany 37: 380-383 doi: 10.1007/BF02904197

[B59] Rasingam L (2012) *Trichosanthes quinquangulata* (Cucurbitaceae) - a new record for India from Andaman Islands. Rheedea 22 (1): 9-10

[B60] Renner SS Schaefer H Kocyan A (2007) Phylogenetics of *Cucumis* (Cucurbitaceae): Cucumber (*C. sativus*) belongs in an Asian/Australian clade far from melon (*C. melo*). BMC Evolutionary Biology 2007, 7: 58. doi: 10.1186/1471-2148-7-58 10.1186/1471-2148-7-58 PMC3225884 17425784

[B61] Sanjur OI Piperno DR Andres TC Wessel-Beaver L (2002) Phylogenetic relationships among domesticated and wild species of *Cucurbita* (Cucurbitaceae) inferred from a mitochondrial gene: Implications for crop plant evolution and areas of origin. Proceedings of the National Academy of Science, USA 99: 535-540 doi: 10.1073/pnas.012577299 10.1073/pnas.012577299 PMC117595 11782554

[B62] Schaefer H Renner SS (2010) A three-genome phylogeny of *Momordica* (Cucurbitaceae) suggests seven returns from dioecy to monoecy and recent long-distance dispersal to Asia. Molecular Phylogenetics and Evolution 54 (2): 553-560 doi: 10.1016/j.ympev.2009.08.006 19686858 10.1016/j.ympev.2009.08.006

[B63] Schaefer H Renner SS (2011a) Cucurbitaceae. In: Kubitzki K (Ed), *Families and Genera of Flowering Plants*. Vol. 10, pp. 112–174. Springer Verlag, Berlin, Germany.

[B64] Schaefer H Renner SS (2011b) Phylogenetic relationships in the order Cucurbitales and a new classification of the gourd family (Cucurbitaceae). Taxon 60 (1): 122-138

[B65] Schaefer H Heibl C Renner SS (2009) Gourds afloat: A dated phylogeny reveals an Asian origin of the gourd family (Cucurbitaceae) and numerous oversea dispersal events. Proceedings of the Royal Society B, 276: 843-851 doi: 10.1098/rspb.2008.1447 19033142 10.1098/rspb.2008.1447 PMC2664369

[B66] Schaefer H Kocyan A Renner SS (2008) *Linnaeosicyos* (Cucurbitaceae): a new genus for *Trichosanthes amara*, the Caribbean sister species of all Sicyoeae. Systematic Botany 33 (2): 349-355 doi: 10.1600/036364408784571707

[B67] Sebastian PM Schaefer H Telford IRH Renner SS (2010) Cucumber and melon have their wild progenitors in India, and the sister species of *Cucumis melo* is from Australia. Proceedings of the National Academy of Science, USA 107 (32): 14269-14273 doi: 10.1073/pnas.1005338107 10.1073/pnas.1005338107 PMC2922565 20656934

[B68] Telford IRH Schaefer H Greuter W Renner SS (2011) A new Australian species of *Luffa* (Cucurbitaceae) and typification of two Australian *Cucumis* names, all based on specimens collected by Ferdinand Mueller in 1856. PhytoKeys 5: 21-29 doi: 10.3897/phytokeys.5.1395 22171190 10.3897/phytokeys.5.1395 PMC3174449

[B69] Volz SM Renner SS (2009) Phylogeography of the ancient Eurasian medicinal plant genus *Bryonia* (Cucurbitaceae) inferred from nuclear and chloroplast sequences. Taxon 58 (2): 550-560

[B70] Wasylikowa K van der Veen M (2004) An archaeobotanical contribution to the history of watermelon, *Citrullus lanatus* (Thunb.) Matsum. & Nakai (syn. *C. vulgaris* Schrad.). Vegetation History and Archaeobotany 13: 213-217 doi: 10.1007/s00334-004-0039-6

